# Cyanochelin B: a cyanobacterium-produced siderophore with photolytic properties that negate iron monopolization in UV light

**DOI:** 10.1128/aem.02566-24

**Published:** 2025-10-15

**Authors:** Berness P. Falcao, Viviana Di Matteo, Pavel Hrouzek, Lenka Štenclová, Petra Urajová, Jan Mareš, Jan Kuta, José Alberto Martínez Yerena, Eliška Kozlíková-Zapomělová, Germana Esposito, Alfonso Mangoni, Valeria Costantino, Tomáš Galica

**Affiliations:** 1 Centre Algatech, Institute of Microbiology of the Czech Academy of Sciences 86863, Třeboň, Czech Republic; 2 Faculty of Science, University of South Bohemia 204738 , Ceske Budejovice, Czech Republic; 3 Department of Pharmacy, Università degli Studi di Napoli Federico II 60240, Napoli, Italy; 4 Biology Centre of the Czech Academy of Sciences, Institute of Hydrobiology 90801, Ceske Budejovice, Czech Republic; 5 Department of Pharmaceutical Biology, Institute of Pharmacy, Freie Universität Berlin 9166, Berlin, Germany; 6 RECETOX, Faculty of Science, Masaryk University 602706, Brno, Czech Republic; University of Illinois Urbana-Champaign, Urbana, Illinois, USA

**Keywords:** siderophores, photolytic, beta-hydroxy-aspartate, secondary metabolites, cyanobacteria, microbial interactions, iron acquisition, structural elucidation, *Leptolyngbyaceae*, co-cultuvation

## Abstract

Siderophores are low-molecular-weight compounds excreted by microorganisms to acquire iron and possibly to monopolize iron resource to achieve competitive advantage over other strains, or to trade for other substrates in mutualistic relationships. Siderophores that employ β-hydroxy-aspartate (β-OH-Asp) for iron chelation can undergo UV-mediated photolytic cleavage, simultaneously reducing Fe^3+^ to Fe^2+^. Photolytic siderophores can promote algal-bacterial mutualism, where the bacteria provide iron in exchange for dissolved organic carbon. We present a comprehensive characterization of cyanochelin B, a photolytic β-OH-Asp-containing siderophore produced by the filamentous cyanobacterium *Leptolyngbya* sp. NIES-3755. Combining nuclear magnetic resonance, high-resolution mass spectrometry, bioinformatic analyses, and Marfey’s and Murata’s method, we elucidated the structure of cyanochelin B, including the configuration of its stereocenters. Cyanochelin B-iron complexes rapidly photolyse under UV light (t_1/2_ = 2.3 min; 19.6 µmol m^−2^ s^−1^ UV-A) and release Fe^2+^. Using a coculture setup with *Leptolyngbya* and *Synechocystis* sp. PCC 6803 (a non-siderophore producer) in membrane-separated compartments and alginate-embedded FeCl_3_ to simulate poorly accessible precipitated iron, we demonstrate cyanochelin B mode of actions. Our results show that in the absence of UV light, cyanochelin B efficiently monopolizes iron, favoring *Leptolyngbya*. However, UV light eliminates this monopolization, making iron available to any cohabiting, also possibly competing, organisms. We further report isolating novel cyanochelin B-producing *Phormidesmis* strains from field material and discuss the broader implications of photolytic siderophores. In conclusion, our interdisciplinary approach led to the discovery of a novel photolytic siderophore, cyanochelin B, and highlighted its possible role in distributing iron in microbial communities.

**IMPORTANCE:**

Iron is an essential micronutrient that is required by all living organisms as a cofactor of indispensable enzymes. Due to its specific properties, it is mostly precipitated and is biologically unavailable. Microbes produce siderophores, low-molecular-weight compounds that bind iron, to facilitate iron uptake. Siderophores are mediators of microbial interactions and facilitate competitive exclusion of non-compatible strains or support mutualistic partners and cheater strains. Here, we adopt an interdisciplinary strategy and report a complete structural elucidation of cyanochelin B, a photolytic cyanobacterial siderophore that contains β-hydroxy-aspartate (β-OH-Asp). Our coculture experiments show that cyanochelin B can either monopolize iron to its producer or make it accessible to other strains, depending on the presence of UV light. Moreover, our data suggest that the benefits from production of photolytic siderophores are not restricted to the producer or cohabiting bacteria but are rather available to the entire irradiated community. Out of the known siderophores, 17.5% contain the photoreactive β-OH-Asp and therefore may play a similar role.

## INTRODUCTION

Cyanobacteria are photoautotrophic prokaryotes that use solar energy to fix inorganic carbon (1). As extremophiles, they often serve as key primary producers in environmental conditions that do not favor land plants or macroscopic algae and provide the surrounding microbial community with fixed organic carbon (2, 3). Although cyanobacteria and heterotrophic bacteria utilize complementary sources of carbon and energy, they often compete for other essential nutrients. Iron is a crucial micronutrient vital to the enzymes of the respiratory chain and the photosynthetic machinery and is therefore required by both phototrophs and heterotrophs alike (4, 5). While iron is the fourth most abundant element in Earth’s crust, its complex chemistry often makes it inaccessible and thus limiting biological productivity in areas like ocean gyres (6, 7).

Many strategies have evolved to cope with iron limitation, such as the accumulation of iron reserves or the production of siderophores—iron-chelating compounds that facilitate iron uptake (5, 8, 9). These siderophores are composed of both iron-chelating residues, such as catecholate, hydroxamate, or carboxylate, and additional structural features, e.g., modified amino or fatty acids, which may help in fine-tuning their functional properties. The combination of these residues has resulted in over 700 unique structures described thus far (10). The repertoire of known cyanobacterial siderophores counts 24 structures in total—schizokinen, six variants of synechobactin, three variants of anachelins, six variants of cyanochelins, three leptochelins (5, 11, 12), and five recently reported variants of lusichelins (13).

Siderophores are released into the environment to scavenge for iron, and once it is bound, the iron-siderophore complexes are taken up and processed by a multi-step transport machinery. Siderophore-producing bacteria possess importers that efficiently recognize and retrieve iron bound by their own siderophore and thus gain access to additional sources of iron. Cells lacking a compatible siderophore importer are locked away from these iron-siderophore complexes and, in time, may be outcompeted (4). However, many non-siderophore-producing microbes can purposefully take up the siderophores produced by others and thus avoid the metabolic costs of siderophore production while attaining their benefit (4, 14). In extreme cases, such microbes depend on specific siderophores produced by other species as their sole means of iron uptake (15). Thus, siderophores limit the access to iron exclusively for the cells with the appropriate transporters, although they may not produce the siderophores themselves.

Many siderophores that bind iron via β-hydroxy-aspartates (β-OH-Asp) undergo lysis upon exposure to UV-A light and, during the process, reduce the bound Fe^3+^ to Fe^2+^ (16–18). Fe^2+^ is more soluble at neutral pH and is easily and non-exclusively taken up by microbial cells (19). Photolytically reduced iron is thought to be a service offered by bacteria to siderophore non-producing eukaryotic algae in return for provided dissolved organic carbon (20, 21). However, this is unlikely to be a relevant explanation for siderophore-producing photoautotrophic cyanobacteria. Siderophores can be a focal point of complex microbial interactions in iron-limited communities.

Previously, we described a novel class of cyanobacterial siderophores named cyanochelins (12). Cyanochelins feature two β-OH-Asp for iron chelation, can perform a photolytic reduction of ferric iron, and, according to our bioinformatic survey, can be expected to be widely distributed. Recently, we have detected cyanochelin B in a field-obtained microbial community that was experimentally starved for iron. In this paper, we take an interdisciplinary approach to provide a comprehensive characterization of cyanochelin B, including its structure, stereochemistry, and photolytic properties. We also investigate the effect of UV-dependent photolysis of iron-cyanochelin complexes on the distribution of iron in a coculture system.

## RESULTS

### Structure of cyanochelin B

**Fig 1:**
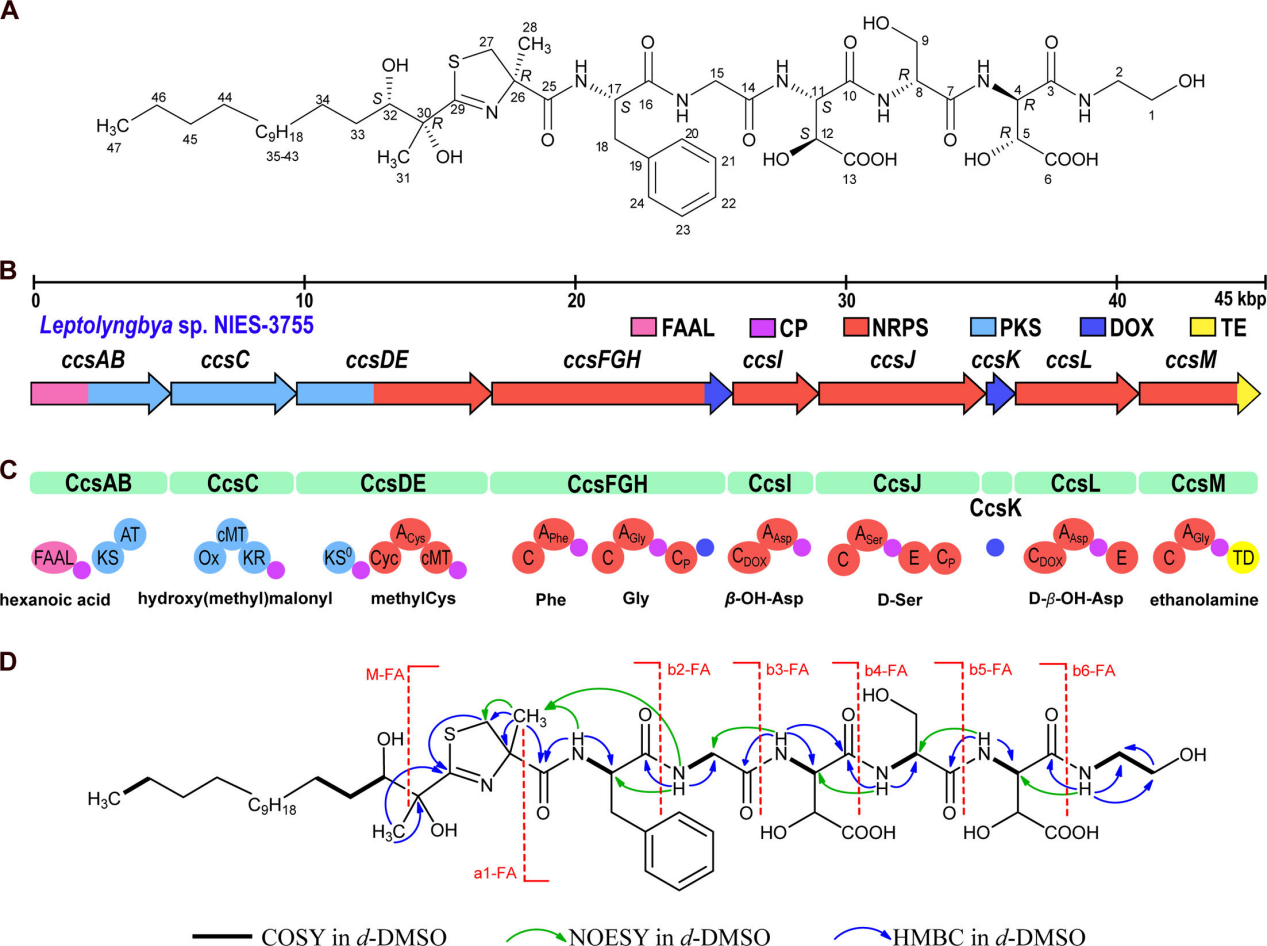
Overview of structural features and biosynthesis of cyanochelin B. (**A**) Structure and stereochemistry of cyanochelin B. (**B**) Architecture of the gene cluster encoding hybrid PKS/NRPS biosynthesis of cyanochelin B (BGC). The BGC is located on plasmid 2 of *Leptolyngbya* sp. NIES-3755, NCBI accession number: AP017310.1, on the complement strand at coordinates 85398-32159. Please note that the BGC spans over beginning/end of the circular sequence. Genes are color-coded according to the type of biosynthetic step: FAAL, fatty acyl-AMP ligase, pink; CP, carrier protein, purple; NRPS, non-ribosomal peptide synthetase, red; PKS, polyketide synthase, light blue; DOX, aspartate β-hydroxylase, blue; TE, thioesterase, yellow. (**C**) Modules of the PKS/NRPS encoded by the cluster: KS, β-ketoacyl synthase; KS0, non-elongating β-ketoacyl synthase; AT, acyltransferase; Ox, flavin-containing monooxygenase; cMT, C-methyltransferase domain; KR, β-ketoacyl reductase; Cyc, cyclization (thiazole-forming) condensation domain; C, condensation domain; A, adenylation domain; E, epimerase domain; TD, thioester reductase domain. Subscript specifies the type of C domains or the specificity of A domains. (**D**) Most relevant 2D NMR correlations and MS fragments of cyanochelin B.

Cyanochelin B (Fig. 1A, 2 mg) at 99% purity was obtained using high-performance liquid chromatography (HPLC) fractionation of a refined biomass extract (for details, see Materials and Methods) from iron-starved *Leptolyngbya* sp. NIES-3755. The purified compound was subjected to a full set of 1D and 2D homo- and hetero-nuclear magnetic resonance (NMR) experiments complemented by high-resolution mass spectrometry analysis (HRMS). Evaluation of cyanochelin B structure was further supported by bioinformatic analysis of its putative biosynthetic gene cluster (BGC) (Fig. 1B and C) and Marfey’s and Murata’s methods for determination of stereochemistry (12, 22, 23).

The HRMS spectrum of cyanochelin B (Fig. S1; Table S1) showed a molecular ion peak [M+H]^+^ at m/z 1,026.5111 corresponding to the molecular formula C_47_H_75_N_7_O_16_S (Fig. S1). Tandem mass spectrometry in combination with collision-induced dissociation was employed to produce fragmentation spectra (MS/MS) of the candidate ion. The MS/MS spectrum of ion m/z 1,026.5111 displayed a characteristic fragment ion at m/z 786.2613, which corresponds to a loss of the N-terminal hydroxylated hydrocarbon chain cleaved between C-30 and C-32, yielding a peptide core of the siderophore (M-FA, Fig. 1D; Fig. S1). Subsequently, the peptide core yields an MS/MS spectrum corresponding to the sequential losses of individual amino acid/residues starting from the C-terminus and forming fragments at 725.2580 (b6-FA, loss of ethanolamine), 594.1889 (b5-FA, loss of β-OH-Asp), 507.1543 (b4-FA, loss of Ser), 376.1325 (b3-FA, loss of β-OH-Asp), 319.1107 (b2-FA, loss of Gly), and 144.0474 (a1-FA, loss of Phe) and was in congruence with the specificity and order of A domains found in the non-ribosomal peptide synthetase-polyketide synthase (NRPS-PKS) BGC located on plasmid 2 of *Leptolyngbya* sp. NIES-3755 (GenBank: AP017310.1, for biosynthesis and MS fragmentation, see supplementary data, Tables S1 and S2; Fig. S1 and S2).

The peptide substructure was elucidated by a full set of NMR data (Table S3; Fig. S3 to S11, for summary, see Fig. 1D), i.e., by the presence of five distinct amide-NH signals (δ_H_-DMSO of NH-17: 7.65 ppm, NH-15: 8.27 ppm, NH-11: 8.07 ppm, NH-8: 7.83 ppm, NH-4: 7.93), four signals related to α-amino acid protons (δ_H_-DMSO of H-4: 4.69 ppm, H-8: 4.45 ppm, H-11: 4.76 ppm, H-17: 4.60 ppm), and two signals related to methylene protons attributable to a glycine residue (δ_H_-DMSO of H-15a: 3.89 ppm and H-15b: 3.80 ppm). Two signals related to methine protons binding oxygen, C-5 and C-12, resonating at δ_H_-DMSO 4.60 ppm and 4.51 ppm, respectively, confirmed the localization of hydroxyl groups on β-carbon of aspartate residues. Finally, the presence of amide signal (δ_H_-DMSO of NH-2: 7.68 ppm) and homonuclear correlation spectroscopy (COSY) and heteronuclear multiple-bond correlation spectroscopy (HMBC) correlations suggest that the C-terminal residue is ethanolamine, presumably derived from a Gly reduced to a primary alcohol by a thioesterase module with reductive function found to be encoded alongside a Gly-specific NRPS module in likely the last gene of the deduced BGC (BAU16060; for details, see Fig. S2).

The ¹H-NMR signals indicated the presence of a saturated hydrocarbon chain. Typical methyl triplet at 0.86 ppm (C-47) is correlated in the COSY spectrum to the methylene resonating at 1.26 ppm (C-46) and is followed by a 14-methylene chain (δ_H_ from 1.23 to 1.51 ppm, δ_C_ from 22.1 to 31.3 ppm). This 15-carbon chain is attached to the methine carbon binding oxygen (δ_H_-32: 3.47 ppm, δ_C_-32: 76 ppm), which is in turn connected to the quaternary carbon (δ_C_-30: 76.8 ppm) that binds an oxygen and a methyl group C-31 (δ_H_-31: 1.34 ppm, δ_C_-31: 24.0 ppm), as is confirmed by the HMBC correlation shown in Fig. 1. The quaternary carbon is linked to a methyl-thiazoline unit, as evidenced by HMBC correlation C-31/C-29 and C-27/C-29. The presence of a methyl-thiazoline residue is expected due to the presence of a predicted cysteine-specific NRPS module with a cyclization domain and a methyltransferase domain (Fig. 1; Fig. S2) and is congruent with the obtained HMBC and nuclear Overhauser effect spectroscopy (NOESY) correlations (Table S3; Fig. S7 and S9) and due to the presence of sulfur also with the isotopic pattern of fragment 144.0474 observed by HRMS.

Cyanochelin B thus consists of [acylated thiazoline^1^, Phe^2^, Gly^3^, β-OH-Asp^4^, Ser^5^, β-OH-Asp^6^, ethanolamine^7^] and contains nine stereocenters (Fig. 1). Marfey’s method was applied to determine the absolute configuration of the α-stereocenters of amino acids and suggested the presence of l-Phe (*S*-Phe), d-Ser (*R*-Ser), and both the l-(2*S*,3*S*) and d-(2*R*,3*R*) forms of *threo* β-OH-Asp (Fig. S12 and S13). The observation aligns with the presence of epimerase domains in NRPS modules responsible for incorporating Ser^5^ and β-OH-Asp^6^. The placement of l- and d-enantiomers of *threo*-β-OH-Asp within cyanochelin B was not apparent at first. However, a careful examination of the NMR data of β-OH-Asp^6^ moiety revealed that the proton H-4, resonating as a double doublet at δ 4.69, is coupled to H-5 with a ^3^*J*_H,H_ coupling constant of 2.3 Hz (Table S3; Fig. S10). Furthermore, a comparison of the multiplicity of H-4 and H-5 in the normal ¹H NMR spectrum and in the 1D sections of the HMBC spectrum taken at δ 173.3 (C-6) and δ 168.8 (C-3), respectively, showed that the ^3^*J*_C,H_ coupling constants between H-4 and C-6 and between H-5 and C-3 are both small (Fig. S10). The combined data enabled the application of Murata’s method, establishing the relative configuration of β-OH-Asp^6^ as *threo*. Similar results were obtained for β-OH-Asp^4^, which also has a *threo* relative configuration (Fig. S11). Bioinformatic analysis of the BGC suggests the same configuration. Reitz and colleagues investigated the stereochemistry of β-OH-Asp in siderophores and reported that with one exception, all β-OH-Asp in siderophores are either d-*threo* or l*-threo* (24). According to their study, the NRPS-fused β-hydroxylases should produce *S,* while the self-standing enzymes should produce *R* configuration at the β-carbon. The NRPS module that should incorporate β-OH-Asp at position 4 lacks an epimerase (CcsI) and is preceded by an NRPS-fused β-hydroxylase (CcsFGH); hence, the amino acid at position four is expected to be l-*threo*-β-OH-Asp (11*S*,12*S*). The NRPS module responsible for the incorporation of amino acid at position 6, CcsL (BAU16061), is Asp-specific, contains an epimerase domain, and is preceded by a self-standing β-hydroxylase (CcsK) and should produce d-*threo* β-OH-Asp (4*R*,5*R*).

As for the configuration at C-26 of thiazoline, a methylcysteine is formed from the opening of the thiazoline ring during the hydrolysis of cyanochelin B. Using Marfey’s method, it was determined that this compound is l-methylcysteine (*R*-methylcysteine). Although we did not have an authentic sample of either d- or l-methylcysteine, we found relevant literature that provided useful information. According to Carmeli et al. (25), the l-methylcysteine-l-DAA derivative exhibits longer retention times than the d-methylcysteine-l-DAA derivative. Therefore, the l-DAA derivative, showing a longer retention time (Fig. S12), must be l-methylcysteine-l-DAA. In comparison, the d-DAA derivative with a shorter retention time must be l-methylcysteine-d-DAA, enantiomeric with d-methylcysteine-l-DAA.

The ketoreductase domain of CcsC (BAU16015) is expected to perform a stereospecific reduction of the β-keto group at C-32 (26). Due to the absence of conserved LDD and HXXY motifs, the reduction of the oxo group at C-32 yields a hydroxyl in the *S* configuration at the respective position (26). The NMR measurements suggest the relative configuration at C-30/C-32 to be *threo* (either 30*S*,32*R* or 30*R*,32*S*) because the multiplicity of H-32, resonating as a broad doublet (*J* = 9.9 Hz), aligns with that reported in the literature for the *threo* stereoisomers of methyl 2,3-dihydroxy-2-methyloctanoate (27, 28). Taking together the specificity analysis of CcsC and the NMR data, we conclude that the configuration at C-30/C-32 of cyanochelin B is a *threo* 30*R* and 32*S*.

### Cyanochelin B is rapidly photolysed in the presence of iron and UV-A light

Siderophores containing a β-OH-Asp in their structure can reduce the bound ferric iron if exposed to UV light with simultaneous photolytic cleavage of the siderophore molecule. To understand how cyanochelin B functions in nature, as well as in our laboratory experiments, we have determined cyanochelin B half-life at different UV light intensities (Table S4) using a source of artificial UV-A light, and also included samples exposed to natural sunlight.

**Fig 2:**
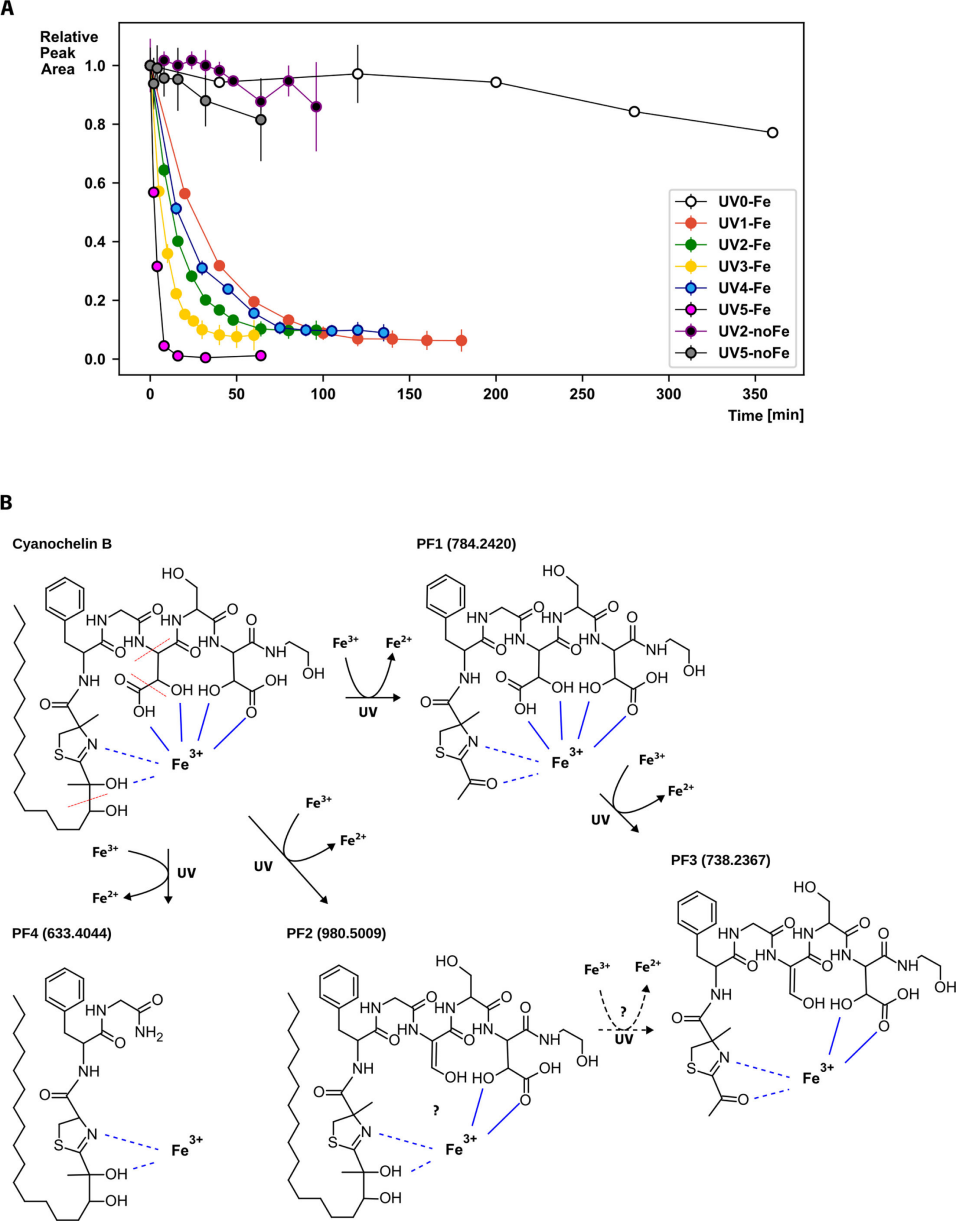
Photolysis of cyanochelin B. (**A**) Kinetics of photolysis of cyanochelin B depicted as decrease in relative peak area calculated from HPLC-HRMS analyses. The concentration of intact cyanochelin was evaluated as peak area of ion with m/z 1,026.5068 and normalized on the average value of time point zero. Each point represents an average value of three replicates with standard deviation indicated as error bars. Color coding according to legend. Intensities of the UV-A light in the individual conditions are indicated in Table 1. (**B**) Scheme of possible photolytic reactions of cyanochelin B and resulting photolytic fragments.

Cyanochelin B showed no or very mild decomposition in the absence of UV light or ferric iron. In the presence of UV light, the iron-siderophore complexes underwent a photolytic reaction with 0-order kinetics and a half-life of 2.3 min at the highest UV irradiation tested (19.6 µmol m^−2^ s^−1^, Table 1; Fig. 2A) and 24.1 min in the light conditions that were later used in the co-cultivation experiments (1/2 UV intensity, including the shading effect of lid of the well plate).

**TABLE 1:** Rates of photolysis of cyanochelin B under different light conditions^*a*^

Conditions	k (min^−1^)	Half-life (min)	*R* ^2^	Time points used for regression	UV-A (315–400 nm) photon flux density (µmol/m^2^/s)	Note
UV1-Fe	0.023	30.3	0.957	6	1.41	UV-LED-Panel at minimum output
UV2-Fe	0.042	16.4	0.970	6	2.53	UV-LED-Panel at ~1/2 output
UV3-Fe	0.078	8.9	0.947	6	4.14	UV-LED-Panel at max output
UV4-Fe	0.029	24.1	0.969	5	2.06	UV2 with lid
UV5-Fe	0.295	2.3	0.952	4	19.62	Natural sunlight 28 April, noon

^*a*^ Photolysis of cyanochelin B-iron complexes under various light conditions was followed by HPLC-MS analysis. The concentration of intact cyanochelin was evaluated as peak area of ion with m/z 1,026.5068 and normalized on the average value of time point zero. The 0-order reaction rates were calculated from indicated number of selected time points, each consisting of three data points. In later time points, the concentration of the monitored ions fell below noise level, and such time points were excluded from the calculations. The normalized data were log-transformed, and analysis of linear regression was performed to produce reaction rates/half-life times. *R*^2 ^values are provided as measure of the reliability of the linear regression. Intensity of applied UV light was measured using SpectraPen (Photon System Instruments).

Further HPLC-HRMS analysis revealed several new compounds—candidate photolytic fragments (PF1–PF4, Fig. 2B; Fig. S14 to S17). Their MS/MS spectra contained signature ions confirming that the compounds originate from cyanochelin B (Fig. S14 to S17) and provided information to identify the site of photolytic cleavage. One cleavage site was between the two hydroxyl groups of the aliphatic chain (C-30/C-32, Fig. 2B), a second one was between the β- and γ-carbons of the β-OH-Asp at position 4 (C-12/C-13), corresponding to photolytic decarboxylation of this residue, and finally, the third one was between Gly and β-OH-Asp at the peptide backbone (C-11/N).

Of the identified photolysis products, the most interesting was photolytic fragment PF1 with m/z 784.2420. A complementary HPLC analysis that preserves iron-siderophore complexes revealed an ion at m/z 837.1538. The observed m/z shows an isotope cluster compatible with the presence of one iron atom and corresponds to PF1 ([M+H]^+^; m/z 784.2420, error 4.3 ppm) with bound iron ([M-2H+Fe]^+^, 837.1538, error 3.7 ppm). According to MS/MS fragmentation, PF1 corresponds to cleavage of the aliphatic chain between carbons 30 and 32 and is accompanied by a reduction of the neighboring hydroxyl to a ketone (Fig. 2B). With two β-OH-Asp residues present in the fragment, the fragment should retain its affinity to iron. It is unclear whether PF1 binds Fe^2+^ or Fe^3+^, but presence of PF3 (m/z 738.2367, error 4.4 ppm) suggests that PF1 (or eventually PF2) could undergo a second photolytic cleavage, upon which the carboxyl group of β-OH-Asp on position 4 is cleaved off (C-12/C-13, see Fig. 2B).

### Cyanochelin B promotes iron starvation of *Synechocystis*

To assess to what degree *Leptolyngbya* can monopolize iron by producing cyanochelin B, it was cultivated along with *Synechocystis* sp. PCC6803 as a reporter strain. *Synechocystis* does not produce its own siderophores and has growth rates and nutrient requirements similar to those of *Leptolyngbya*. First, we needed to verify that cyanochelin B has no additional inhibitory effect besides iron deprivation on *Synechocystis*.

**Fig 3:**
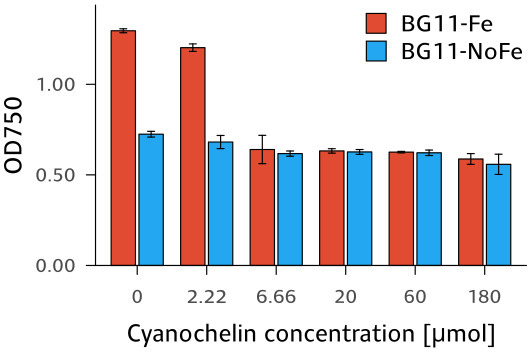
Endpoint OD of *Synechocystis* grown for 10 days in the presence of cyanochelin B in standard BG-11 and its iron-deprived version. Each bar represents the mean value of three replicates with standard deviation as error bars.

The cultures of *Synechocystis* in standard and iron-deprived media were exposed to cyanochelin B. Most importantly, the concentration of cyanochelin B had minimal to no impact on the culture grown in iron-deprived media (Fig. 3). For example, at 0 µM and 180 µM final cyanochelin B concentration, the optical density at 750 nm (OD) of *Synechocystis* increased from 0.2 to 0.65 and 0.6, respectively, indicating negligible inhibitory effect. The cultures grown in standard medium treated with cyanochelin B up to 2.2 µM reached approximately 1.5 times higher densities than those in iron-deprived media. In contrast, *Synechocystis* grown with cyanochelin B concentrations higher than 6 µM reached roughly the same density as their counterparts in iron-deprived media. The results show that cyanochelin B’s primary inhibitory mechanism involves preventing *Synechocystis* from acquiring iron, becoming effective once the concentration of cyanochelin B exceeds that of iron. The observation that higher cyanochelin B levels did not further reduce growth confirms that its impact is solely due to iron sequestration, not additional toxicity. It should be noted that despite aiming for ~10 µM of available Fe in the assay medium (see Materials and Methods), iron precipitation in BG-11 might have meant a lower actual biologically available iron concentration, potentially leading to full iron titration by just 6.7 µM of cyanochelin B.

### UV-A light abolishes iron monopolization by cyanochelin B

Cyanochelin-iron complexes are not accessible to *Synechocystis*; however, it can access the free iron released as a result of their photolysis and benefit from *Leptolyngbya*-produced cyanochelins. To verify this, an experimental coculture with the presence of UV light (and non-UV as control) was carried out.

Initially, we evaluated the growth of the selected strains under our experimental conditions individually. Both *Leptolynbgya* and *Synechocystis* monocultures were inoculated at identical OD (0.02) and grown for 14 days. In standard BG-11 medium, *Leptolyngbya* grew well and reached final ODs 1.48 (noUV) and 1.64 (UV) (Fig. S18). Compared to *Leptolyngbya*, *Synechocystis* exhibited even better growth, OD 2.74, in standard BG-11 and UV, as well as in noUV to OD 1.68. In iron-deprived conditions, *Leptolyngbya* reached OD 0.54 and 0.71 for non-UV and UV conditions, and *Synechocystis* reached OD 0.99 and 0.82 in non-UV and UV conditions, respectively. Despite pre-starvation, cells of both strains in iron-deprived media underwent approximately five cell divisions (considering starting OD of 0.02), indicating that they either retained some reserves or were able to scavenge residual iron from the media.

In monocultures with an immobilized source of iron, OD of the *Leptolyngbya* culture increased to 0.89 and 1.05 for non-UV and UV, respectively. This suggests that the producer takes up the siderophore-bound and photolytically reduced iron with comparable/equal efficiency. To a certain extent, *Synechocystis* was also able to benefit from the presence of bead-enclosed particulate iron. Complementary inductively coupled plasma mass spectrometry (ICP-MS) measurements estimated that an average alginate bead contains 2.08 ± 0.21 µg Fe (mean ± SD, *n* = 9) and that our iron-deprived medium contained 1.43 ± 0.23 µg/L (mean ± SD, *n* = 3), which corresponds to 700 times lower Fe content in comparison to standard BG-11 (~1 mg/L). Importantly, 3 mL of iron-deprived BG-11 incubated with three alginate beads for 13 days exhibited a concentration of 12.47 ± 0.12 µg/L Fe (mean ± SD, *n* = 3), accounting for less than 35 ng of iron. The amount of the leaked Fe is negligible compared to the amount of iron in the bead (~0.017%); however, it is still ~10× more free iron than what is available in the iron-deprived media. Components of the BG-11 media, particularly citrate and ethylenediaminetetraacetic acid, exhibit a certain affinity to iron and could possibly facilitate the observed leakage of a minor amount of iron. Even if both strains were able to benefit from the immobilized iron bead to a certain extent, the effect of the siderophore is expected to show in coculture where the siderophore from the producer should primarily monopolize or release the iron from the bead.

In coculture, with or without UV in standard BG-11, iron is in excess, and there is no competition for it. At this condition, *Synechocystis* outcompeted *Leptolyngbya*. Perhaps the limitation by other elements such as phosphorus or CO_2_ may hinder *Leptolyngbya* in coculture and make *Synechocystis* the better competitor. In cocultures, with no presence of iron, as expected, both strains grew poorly.

**Fig 4:**
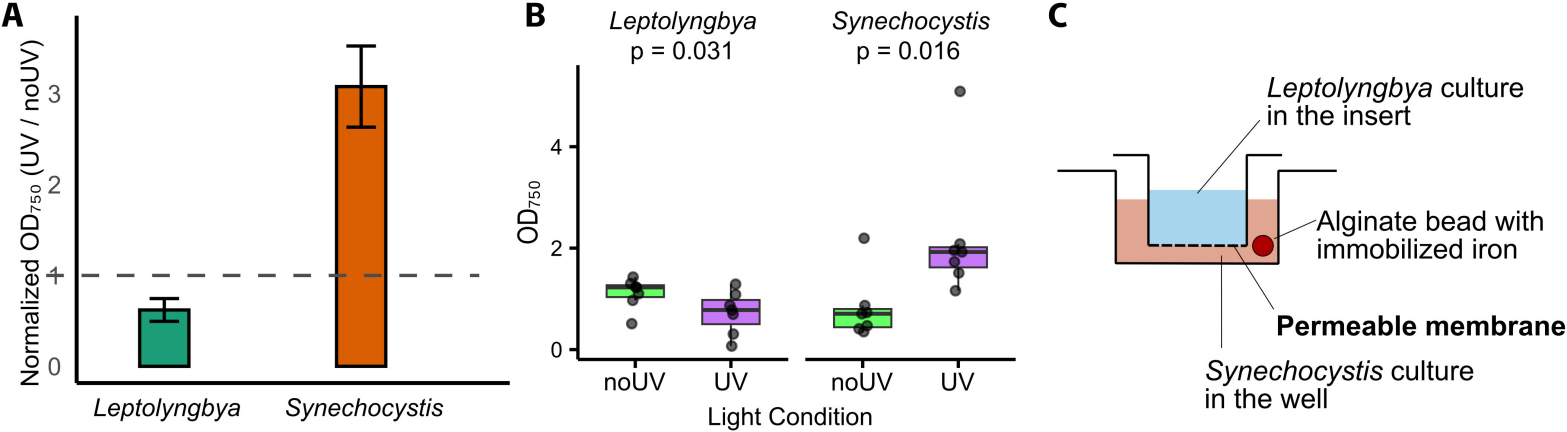
Co-cultivation of *Leptolyngbya* and *Synechocystis*. (**A**) Endpoint ODs of *Leptolyngbya* and *Synechocystis* grown in the presence of UV-A light normalized on their non-UV parallels. The dotted line indicates the noUV readouts normalized to 1 for comparison with the UV conditions. (**B**) Endpoint OD box plots for *Leptolyngbya* and *Synechocystis* in UV and no-UV conditions. Each box plot represents the interquartile range (IQR), the circles indicate individual data points, the thick horizontal line is the median, and the whiskers highlight the range of the data. (**C**) Schematic cross-section of the employed experimental setup, depicting the relative position of the cyanobacteria in the individual compartments and the position of the alginate bead with the enclosed particulate iron.

To assess the growth benefits for *Synechocystis* resulting from cyanochelin B photolysis, we have further focused on the coculture in conditions with bead-immobilized Fe and performed three independent biological replicates. To properly evaluate the results, OD values obtained under UV conditions were normalized to the OD values in the non-UV conditions for both of the strains. We observed that *Leptolyngbya* exhibited a mean fold reduction of approximately 1.61 under UV compared to noUV conditions. In contrast, *Synechocystis* showed a mean fold increase of 3.1 under UV conditions, when compared to noUV. This result strongly indicates that the degree of monopolization of iron by *Leptolyngbya* is reduced under UV light. Additionally, it also suggests that cyanochelin B releases free iron and supports the non-producer strain present in its vicinity (Fig. 4A). For clear representation of individual data points and to verify that the data are statistically significant, the data are also represented as a box plot, and growth of the strains across the seven replicates was compared between the light conditions (Fig. 4B). Both strains showed statistically significant differences between the non-UV and UV conditions*—Synechocystis* (W = 28, *n* = 7, *P* = 0.016, *r* = 0.91); *Leptolyngbya* (W = 1, *n* = 7, *P* = 0.031, *r* = 0.81). Although there are many factors that influence the interaction between these two organisms, it is evident that when they are in close proximity to each other, the photolysis of cyanochelin can alter the dynamics of iron uptake and facilitate the growth of a non-producer by reducing iron monopolization.

### BGCs encoding cyanochelin B production are found in several phylogenetic lineages of cyanobacteria

**Fig 5:**
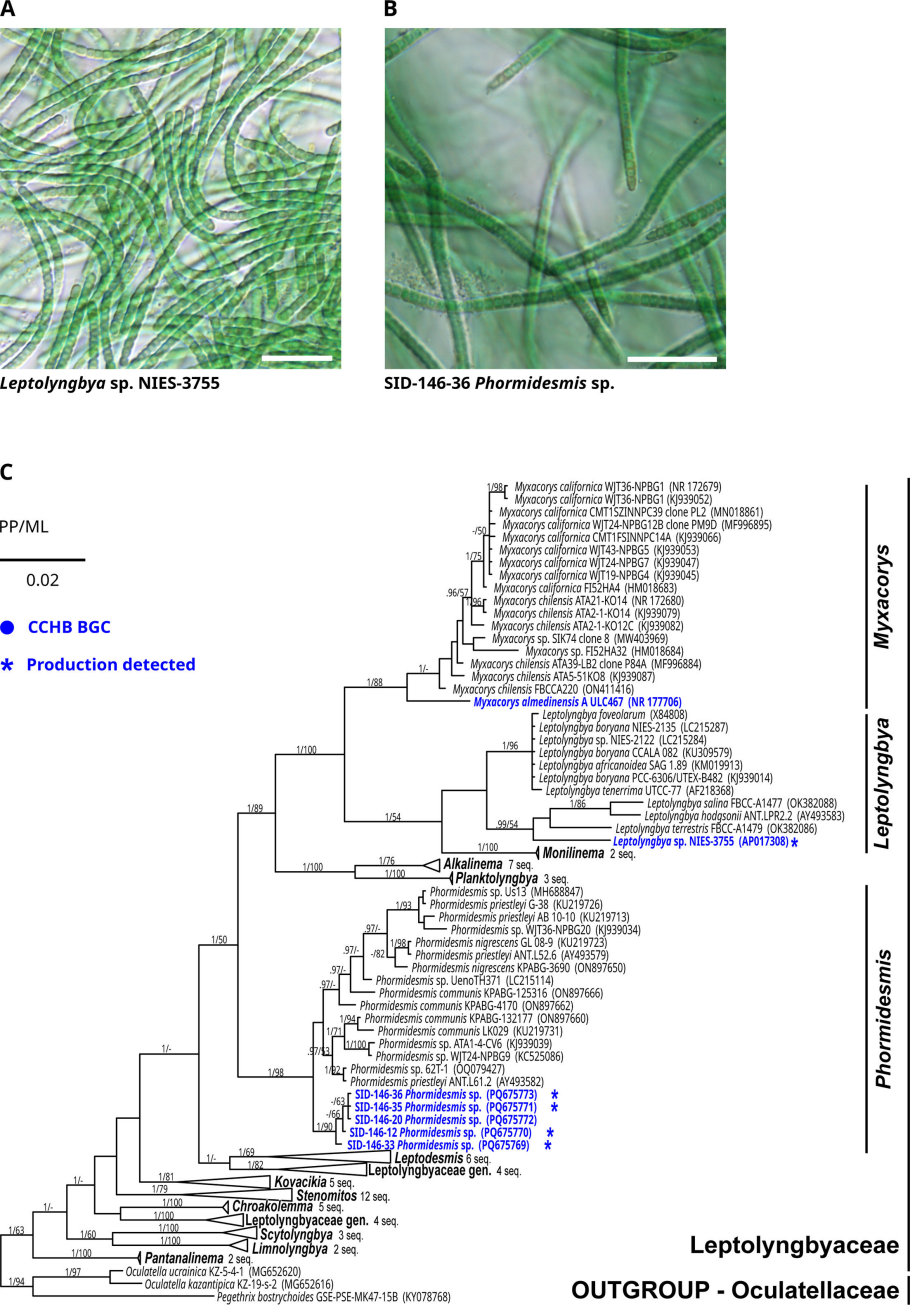
Morphology and phylogeny of cyanochelin B producers. (**A and B**) Microscopic view of the strains investigated in the present study. (**C**) Phylogenetic tree of 16S rRNA gene sequences featuring *Leptolyngbyaceae* taxa hosting biosynthetic gene clusters for cyanochelin B in their genomes. The production of cyanochelin B, which was detected by HPLC-MS, is marked by an asterisk. The topology of the tree represents Bayesian inference analysis. Supports of the branches, posterior probability (PP), and nonparametric bootstrap maximum likelihood (ML) are presented. Scale bars represent 20 µm.

To understand the role of β-OH-Asp siderophores in natural cyanobacterial communities, it is important to first identify the type of habitats in which they occur and then obtain the producers along with additional representatives of their microbial communities. During a broader field survey aimed at sampling of potentially iron-limited cyanobacterial communities that could produce novel siderophores, we obtained material that, after cultivation in iron-deprived media, contained cyanochelin B. Environmental sample number 146 was found as a microbial mat growing on a small wooden bridge within the splash zone of a waterfall in a calcareous region of inland Croatia (Fig. S19). Multiple cyanobacterial morphotaxa were identified in the original sample using light microscopy: *Microcoleus* sp., *Coleofasciculus* sp., *Calothrix* sp., and several *Leptolyngbyaceae* morphotypes. The sample was brought to the laboratory and kept in iron-depleted medium for approximately 3 months until the production of siderophores was induced, and the HPLC-HRMS analysis confirmed the presence of cyanochelin B. During cultivation and iron starvation, four *Leptolyngbya*-like morphotypes prevailed in the batch culture, of which single filaments were picked to isolate clonal strains (Table S5). PCR amplification of three different custom-designed loci (Table S6) across the cyanochelin B BGC was used to identify tentative producer strains. Three PCR-positive strains exhibited identical morphology corresponding to *Phormidesmis* sp. (Fig. 5); therefore, two additional strains of the morphotype were further examined. These isolates were cultivated in iron-depleted media, and the production of cyanochelin B was confirmed by HPLC-HRMS in four of them (S146-12, S146-33, S146-35, S146-36). The analysis of the metagenome and isolation of heterotrophic bacteria from preserved material is currently ongoing.

Genomes of all five isolated cyanobacterial strains were sequenced, and four of these were successfully assembled (JBJPHS000000000, JBJPHT000000000, JBJPHU000000000, and JBJPHV000000000). The genome assembly of strain S146-36 failed due to contamination. The sequenced strains were searched for BGCs homologous to the cyanochelin B BGC as found in *Leptolyngbya* sp. NIES-3755. We confirmed the presence of cyanochelin B-compatible BGC in the three cyanochelin B-producing isolates of *Phormidesmis* isolates, S146-12, S146-33, and S146-35 (Fig. S20), but also in the strain S146-20, in which we could not confirm the production using HPLC-HRMS.

An additional BLAST search against the NCBI database identified a BGC highly similar to that of *Leptolyngbya* sp. NIES-3755 in the genome of *Myxacorys almedinensis* strain A (ULC467) (Fig. S20). Analysis of NRPS adenylation domains (A domains) (Table S2) indicated that this strain is likely a cyanochelin B producer; however, its production capability was not investigated in the current study. The NRPS-PKS biosynthetic core of all analyzed BGCs was organized identically and consisted of nine genes spanning approximately 45 kbp, ordered co-linearly with the predicted biosynthesis (Fig. S2). Details of the BGC organization and encoded NRPS-PKS domains, including the predicted substrate specificity of A domains, were previously described by Galica et al. (12) for *Leptolyngbya* sp. NIES-3755, and are complemented here for *Phormidesmis* sp. 146 isolates and *M. almedinensis* A (Fig. S2; Table S2).

All examined *Phormidesmis* sp. strains’ rRNA operon genes were almost identical, diverging only 0%–0.3% in the 16S rRNA gene and 0%–0.7% in the rRNA internal transcribed spacer (ITS) sequences, suggesting that all are representatives of a single species (29). The cyanochelin B BGCs from the novel strains of *Phormidesmis* sp. represented three different genotypes with an average pairwise nucleotide identity of about 96% across the BGC. The sequence identity between the homologous proteins encoded in the BGC ranged from 93 to 100%.

The phylogenetic analysis of the 16S rRNA gene sequences from all three cyanochelin B-producing taxa and representatives of *Leptolyngbyaceae* did not support the existence of a separate clade with cyanochelin B producers. While all of them were resolved as members of a single family, each of the three taxa harboring the BGC belonged to different genera, separated by a relatively long evolutionary distance (Fig. 5). The average pairwise nucleotide identity across the BGCs was slightly higher between *Leptolyngbya* sp. NIES-3755 and *M. almedinensis* A (90.6%) than between these two strains and *Phormidesmis* sp. (86.9%–88.9%), which was also reflected in their average protein sequence identities (91.3% versus 87.7%–89.4%). The difference was most prominent in the adjacent siderophore transporter gene cassette between *Phormidesmis* and the other two taxa (Fig. S20). In comparison to the set of nine genes presumably involved in cyanochelin B export/import in *Leptolyngbya* sp. NIES-3755 (12) and *M. almedinensis* A (30), the *Phormidesmis* sp. isolates lacked three terminal genes, identified as homologs to DevA, DevB, and DevC, which encode an ABC transport system to possibly export the siderophore from cell interior to periplasmic space. However, a major facilitator protein (cctC–BAU16019) that is also possibly performing the same function is present in all the strains, and these three genes may just code a functionally redundant system.

## DISCUSSION

Siderophores are likely to play a crucial role in the dynamics of microbial communities, as iron deprivation is widespread under our oxidizing atmosphere (19). A substantial body of literature and experimental data show that the exclusive monopolization of iron by siderophore-producing organisms is not the prevalent scenario in complex microbial consortia. Instead, various forms of siderophore piracy and dependencies have been reported (15, 31). The photolytic properties of β-OH-Asp siderophores introduce an additional layer of complexity, since they can support the growth of organisms who are devoid of the proper importing mechanisms, by creating a surplus of Fe^2+^ in the producer’s vicinity (17, 20).

Siderophores are thought to be exchanged for dissolved organic carbon in a mutualistic partnership of algae and bacteria (20, 21). However, cyanobacteria as siderophore producers that can perform photosynthesis to fix organic carbon from light and CO_2_ can challenge this hypothesis. In this context, exploring which organisms are supported by cyanobacterial siderophores and what benefits cyanobacteria may gain in return is interesting. In this present paper, we report our initial steps to establish an experimental model for exploring siderophore-mediated interactions between cyanobacteria and their associated bacteria.

Our experimental setup employs particulate iron immobilized in alginate beads, membrane separation of the strains, and lytic UV-A light (Fig. 4C). We assume that in the environment, iron is often not solubilized but present within a range of centimeters in some particulate form. By enclosing particulate iron in alginate beads, we tried to replicate such conditions - while the chemically inert membrane separates the strains while allowing diffusion. Importantly, the bead was placed in the compartment with the siderophore non-producing strain to ensure that these compounds would cross the membrane on its way to the iron source. Microbial communities often exhibit complex spatial organization with overlapping distribution of individual strains across multiple gradients (1). The lack of spatial organization should not considerably affect our observation of siderophore routing of iron. In the experiments, we have used constant light composed of warm-white light and UV-A provided by LED strips. Reproducing natural light with all its components and variations in a lab is impossible. However, it was important to include a light that would cause photolysis of cyanochelin B, which we discuss below.

Cyanochelin B is an amphiphilic lipo-heptapeptide that employs two β-OH-Asp residues and likely a combination of hydroxyls and a thiazoline ring for iron chelation. This unique structure contributes to the repertoire of known siderophores from cyanobacteria. β-OH-Asp is broadly used as an iron-chelating residue and is found in 124 of 707 (17.5%) siderophores cataloged in a recently updated siderite database (10). The frequent occurrence of β-OH-Asp in siderophores suggests that photolysis of siderophores may be a widespread and currently underestimated phenomenon in microbial ecology. In cyanochelin B, the two β-OH-Asp residues account for four out of six electron pairs required for chelation of ferric iron. Currently, we lack experimental data to unambiguously determine the origin of the remaining two electron pairs. However, on the N-terminal moiety of the peptide, there is a thiazoline linked to a hydroxylated acyl chain. The relative position of the nitrogen in the thiazoline and hydroxyl group on the α-carbon of the acyl chain may provide the required electron pairs, similarly to hydroxyphenylthiazoline found in yersiniabactin or alpha-hydroxyimidazole present in corrugatin (32–34). Furthermore, photolytic cleavage typically occurs near the iron-binding residues (16); hence, the observed cleavage at C-30–C-32 suggests that at least one of the hydroxyls in its proximity is in contact with iron. Confirming the role of these hydroxyls in iron chelation was beyond the scope of this study; however, if it was confirmed by NMR data, it would be a novel structural motif employed for iron chelation.

Photolysis of cyanochelin produces several types of fragments. Their relative abundance cannot be accurately determined due to possibly different ionization at the MS source and lack of analytical standards for individual compounds. However, assuming the same efficiency of MS ionization for all the fragments, it seems that PF1 and PF2 are the most abundant. Interestingly, at least one of the fragments, particularly PF1, seems to retain the ability to chelate iron, similarly as was reported for pacifibactin, petrobactin, aerobactin, and aquachelin (16–18). Less explored is the possible capability of a photolytic fragment to perform an additional round of photolysis. Our data suggest that PF1 can undergo secondary photolysis. However, the fragment must be isolated and tested separately to properly prove it. Although such a capability was considered, e.g., aquachelin, we are currently unaware of any case described.

Cyanochelin B reduces ferric iron to its ferrous form. The measured rates and half-lives of the iron-siderophore complex (maximal rate 0.295 min^−1^ or 17.7 h^−1^; t_1/2_ ~2.3 min) suggest that once iron is bound by the siderophore, it immediately undergoes photoreduction within the range of minutes. Amin and colleagues reported photolytic rates of vibrioferrin (0.031 h^−1^), petrobactin (0.003 h^−1^), and marinobactin (>0.001 h^−1^) illuminated by fluorescent light with photon flux density (PFD) 80 µmol m^−2^ s^−1^ (35). The authors also measured photolysis of vibrioferrin under attenuated sunlight with PFD 500 µmol m^−2^ s^−1^ to later multiply it by 4 (12.9 h^−1^, t_1/2_ ~3.2 min) in order to compare it with previously reported rates of photolysis of aquachelin under sunlight in 2,000 µmol m^−2^ s^−1^ (0.6 h^−1^). Comparing the rates of photolysis of individual siderophores is complicated. First, for the structural differences: petrobactin and vibrioferrin both use a citrate moiety; however, the former is at a central position and the latter is at a terminal position. Marinobactin employs β-OH-Asp that is further cyclized to a nine-membered heterocycle, while aquachelin uses a simple β-OH-Asp. These differences affect the absorbance spectra as well as the possible course of reaction. Second, the spectra of the used light source are not usually available; hence, in combination with different absorption spectra of the siderophores, it is not possible to clearly state how much of the photolytic light is being applied. For UV-dependent photolysis, we have used UV-A emitting LED lights that have an emission peak at 360 nm and photon flux density of 1.4, 2.5, and 4.1 µmol m^−2^ s^−1^ over the range 315 nm–400 nm, and also natural sunlight at an intensity of 19.62 µmol m^−2^ s^−1^ (Fig. S21A; Table S4). Even in UV2 (2.5 µmol m^−2^ s^−1^), which was used for cultivation, the rates for cyanochelin B are higher than for aquachelin in direct sunlight and 36% of the calculated values for vibrioferrin. To obtain more environmentally relevant data and to see how fast the degradation can get, we exposed a mixture of iron and cyanochelin B to natural sunlight. Intense direct sunlight (19.6 µmol m^−2^ s^−1^ over the range 315–400 nm) accelerates the rate of cyanochelin B photolysis to 0.295 min^−1^ or 17.7 h^−1^, which is the highest rate of photolysis of a siderophore measured thus far. Overall, it can be concluded that cyanochelin undergoes photolysis at high rates and that the conditions we have used in the lab are likely to result in photolysis rates comparable to a cloudy day or a morning/evening time of the day (Table 1; Table S4; Fig. S21). In cultivation with bead-enclosed iron, the rate of photolysis is also influenced by the kinetics of formation of iron-siderophore complexes. In nature, the process is likely further complicated by the changing light conditions and much more complex chemical milieu that often includes UV-protective pigments (1). Also, we have used constant light and did not include “night” in our cultivation experiments, during which the pool of cyanochelins could possibly replenish and direct the flow of iron to a different set of microorganisms within the community.

Siderophores are frequently considered to be involved in complex microbial interactions (4). The possible role of photolytic siderophores in microbial communities has been considered previously, however, in the context of the relationship between phototrophic algae and siderophore-producing bacteria (20). In such a case, the exchange of fixed organic carbon for siderophore-mediated access to additional iron sources seems to be a mutually beneficial trade. In our experimental model, we employ two phototrophic organisms that compete for the same set of nutrients and of which only one can produce siderophores. *Synechocystis*, the non-producer, outcompetes the siderophore-producing *Leptolyngbya* in standard cultivation media. Such a situation is unlikely to occur in the alga-bacteria system, where bacteria live on algal exudates. When our model strains are set to compete for a limited source of iron, *Leptolyngbya* is clearly able to monopolize access to it in the absence of UV light. In the presence of UV, however, the photoreduced iron is also available to *Synechocystis* in amounts sufficient to regain its advantage. In the presence of UV light, *Leptolyngbya* clearly supports its direct competitor and is likely to become an indispensable minority in the model system. This is in accordance with the postulates of the Black Queen Hypothesis, which expects the occurrence of strains, so-called “leaky helpers” (*Leptolyngbya*), that form a minority but are retained since they carry the load of an essential metabolic capability crucial for the survival of fast-growing “beneficiaries” (e.g., *Synechocystis*) and of the whole community (31).

Cyanochelin B was detected in iron-starved cultures of *Leptolyngbya* sp. NIES-3755 (36) and in four strains of *Phormidesmis* described in the current study. In addition, cyanochelin B BGCs were found in one more strain of *Phormidesmis* and in *Myxacorys almediensis*. All the concerned strains are simple filamentous cyanobacteria from the *Leptolyngbyaceae* family *sensu* (37, 37, 38). They are found worldwide to grow subaerophytically on exposed soil (*Leptolyngbya,* 36) but also on man-made structures, such as stony walls (*Myxacorys,* [30]) and wooden bridges (*Phormidesmis,* current study). Subaerophytic microbial mats, however, often lack reliable access to required nutrients and face severe fluctuations of light intensity, temperature, and water availability. Such mats are frequently found and are ecologically important in extreme environments, such as deserts, high altitude mountains, and polar regions. It is tough to address the availability of iron for microbial mats. Frequent changes of environmental conditions and possibly steep gradients of pH and redox potential within the microbial mats make it almost impossible to estimate when and whether a particular filament is iron-limited. However, it is reasonable to estimate the cyanobacteria in microbial mats will undergo at least intermittent periods of iron deprivation. In the exposed microbial mats, filamentous cyanobacteria, such as *Leptolyngbya* or *Phormidesmis,* are frequently the keystone species. They provide fixed carbon, possibly also nitrogen, and their filamentous bodies are important structural features that provide shelter for a multitude of associated microbes (2, 3). In our hands, however, the cyanochelin-producing species was not dominant and only rose to prominence during prolonged cultivation in iron-deprived medium and in the absence of UV light.

The strains with cyanochelin B-encoding BGC all belong to a sub-lineage of *Leptolyngbyaceae* consisting of the genera *Leptolyngbya*, *Myxacorys*, *Monilinema*, *Alkalinema*, *Planktolyngbya,* and *Phormidesmis* (38). The cyanochelin BGC organization (Fig. S20) and sequence identity patterns between the individual biosynthetic genes correspond with the reconstructed evolutionary distances between the three taxa (Fig. 5). The metabolic capability to synthesize cyanochelin B could be an ancestral trait, although that would require a high number of gene loss events during which the BGC is compromised. The Black Queen Hypothesis postulates that such events may be frequent, if there is another member of the microbial community to compensate for the lost metabolic capability (14, 15, 31). The concerned phylogenetic lineage, however, includes multiple other strains, i.e., *Leptolyngbya boryana* NIES-2135, *Myxacorys chilensis,* and *Myxacorys californica,* that harbor BGCs that could possibly encode biosynthesis of cyanochelin-like siderophores with two β-OH-Asp included in their structures (12, 38). The situation around the evolution of cyanochelin B BGC is further complicated by the fact that in *Leptolyngbya* sp. NIES-3755, the cyanochelin BGC is located on a plasmid (12) and can thus easily undergo horizontal gene transfer.

Considering the frequency of β-OH-Asp found in siderophores, the photolysis of iron-siderophore complexes and accompanied release of reduced iron may be a phenomenon with underestimated effect on cycling of iron in microbial communities and potentially on global Fe cycle. Cyanochelin B is an example of such a siderophore. It is found across the cosmopolitan *Leptolyngbyaceae* family, and in our test system, it provides reduced iron that non-selectively supports cohabiting organisms, even though the organism is the producer’s competitor.

## MATERIALS AND METHODS

### Strains and cultivation

*Leptolyngbya* sp. strain NIES-3755 was obtained from NIES culture collection and grown on BG-11 medium at 21°C and continuous dispersed light. For the production of the compound, the strain underwent several passages in iron-depleted medium until siderophore production was confirmed by HPLC-HRMS and then harvested. *Synechocystis* sp. PCC 6803 Nixon was kindly provided by Prof. Roman Sobotka (Centre Algatech, Institute of Microbiology of the Czech Academy of Sciences) and kept at 28°C.

Iron-deprived media was prepared analogously to standard BG-11 but without the addition of citrate ammonium iron(III), Brown (Thermo Scientific Chemicals, cat. no. A11199.30). The pH of BG-11 media was adjusted to 7.5 (39).

### Siderophore inhibition test

*Synechocystis* was grown for ~7 days in iron-deprived media at 25°C and 50 µmol m^−2^ s^−1^ with constant bubbling until it reached OD_750_ = 0.8 (1 cm optical path, V-1200 Spectrophotometer, VWR). The culture was spun down by centrifugation (3,000 rpm for 5 min, benchtop centrifuge, Eppendorf). The cell pellets were rinsed by ironless medium, pelleted again, and finally resuspended in both standard and ironless media to final OD_750_ = 0.4. The cell suspensions were used to inoculate a 96-well plate. The treatment solution consisting of ironless medium and cyanochelin B at various concentrations was added to the cell suspension in a 1:1 ratio, yielding cultures with a starting OD_750_ = 0.2 and cyanochelin B concentrations of 180, 60, 20, 6.7, 2.2, and 0 µM. The experiment was evaluated after 10 days of cultivation at 50 µmol m^−2^ s^−1^ and 25°C by measuring OD_750_ using an Omega Fluostar plate reader.

### Preparation of alginate beads

To prepare alginate-enclosed precipitate iron we used sodium alginate [(C₆H₇O₆Na)_n_, cat. no. A3249, PanReac AppliChem], iron(III) chloride hexahydrate (FeCl_3_·6H₂O, analytical grade, cat. no. 16900-30500, PENTA s.r.o.), sodium hydroxide (NaOH, pellets, analytical grade, cat. no. 15760-31000, PENTA s.r.o.) and calcium chloride dihydrate (CaCl_2_·2 H_2_O, reagent grade, ≥99.0%, cat. no. C3881, Honeywell Research Chemicals). An aqueous solution of ferric chloride (0.5M) was precipitated by the addition of 1M NaOH in 1:9 ratio and pelleted by centrifugation. The supernatant was removed, and the pelleted iron precipitates were mixed with aqueous solution of sodium alginate to a final 1% (wt/wt) alginate. The suspension was dropped into a continuously stirred solution of calcium chloride (0.1M) to produce ~40 µL beads, each containing roughly 2.15 µg Fe.

### Iron determination

Determination of iron in alginate beads/scads and cultivation media was performed by ICP-MS. An Agilent 8900 ICP-MS/MS instrument equipped with MicroMist concentric nebulizer, 1Scott double-pass spray chamber, quartz torch with 2.5 mm i.d. injector, and nickel sampling/skimmer cones was used for quantification of iron in digested (beads and scads) or diluted (media) material. ICP-MS/MS was operated at 1,550 W input power, 8 mm sampling depth, and 1.20 L/min flow of carrier gas (Ar). The octapole reaction system, operated in collision mode with helium flow of 10 mL/min, was used for suppression of spectral interferences.

Prior to analysis, alginate beads and scads were mineralized with 1 mL of concentrated nitric acid (HNO_3_, 65%, Suprapur grade, Merck, cat. no. 100441) and 0.5 mL of hydrogen peroxide (H_2_O_2_, 30  % wt/wt in H_2_O, EMSURE grade, Perhydrol for analysis, Merck KGaA [MilliporeSigma], cat. no. 107209 ). Samples were digested at 170°C for 20 min with ramping of temperature in 20 min using microwave decomposition system (MARS 6, CEM Corporation). Finally, mineralized samples were diluted to 10 mL with Milli-Q type 1 ultrapure water (Milli-Q Direct, Milliore). Cultivation media were 5× diluted to 1% nitric acid prior to analysis.

### Co-cultivation experiments

*Leptolyngbya* and *Synechocystis* were cultivated in membrane-separated compartments as monocultures and cocultures (ThinCerts, Greiner, item no. 657641). The cultures were either deprived of iron or the iron was provided as soluble ferric ammonium citrate (~20 µM Fe, standard BG-11) for controls. Initially, an experimental setup was carried out with all these controls and the test. For the test, iron was provided in the form of ferric chloride precipitates enclosed in alginate beads, referred to as bead-immobilized iron, which is supposedly accessible by siderophores. Three biological replicates of the tests were performed. For statistical analysis of the test, the Wilcoxon signed-rank test was applied to each test pair (Strain: UV vs non-UV), and the medians between the UV and non-UV groups were compared using R v.4.4.1 (40).

The cultures were first primed in full iron BG-11 medium, followed by a period of iron starvation for a week. Before plating, *Leptolyngbya* was checked for siderophore production using Chrome Azurol S (CAS) assay (41). Pre-starved cultures of cyanobacteria were inoculated into six-well plates at an initial OD_750_ = 0.05 (1 cm optical path, V-1200 Spectrophotometer, VWR). *Leptolyngbya* was inoculated into the insert compartment, and *Synechocystis* was placed below the insert into the well. The inserts were also added to monocultures to avoid any shading bias. Plates were placed in UV (2.5 µmol m^−2^ s^−1^, UV-A, 315 nm–400 nm) and non-UV conditions to determine the effect of cyanochelin B photolysis. The plates were incubated for 15 to 20 days at 25°C with shaking and continuous light (warm white LED) at 50 µmol m^−2^ s^−1^. At the end of the experiment, the cultures were thoroughly mixed, and their OD_750_ was measured on a V-1200 Spectrophotometer (VWR) in a 1 mL disposable plastic cuvette with a 1 cm optical path.

### HPLC-HRMS analysis

The concentration of cyanochelin B and its photolytic fragments was analyzed on Dionex Ultimate 3000 HPLC system coupled to Bruker Impact HD II mass spectrometer equipped with electrospray ionization. Chromatographic separation was achieved on Waters MaxPeak XBridge Premier BEH C18, 130 Å, 2.5 µm; 50 × 2.1 mm column. Acetonitrile (A) (≥99.95%, HiPerSolv CHROMANORM, VWR Chemicals BDH, Avantor, cat. no. 83639.320) and water (B) (VWR Chemicals BDH, Avantor, cat. no. 83645.320), supplemented with trifluoroacetic acid (≥99.0%, CHROMASOLV for HPLC, Fluka, Avantor, cat. no. 302031) at concentrations of 0.002% and 0.1% vol/vol, were used as mobile phases. The column was eluted with the following gradient: 0 min 5/95%; 2 min 5/95%, 8 min 100/0%, 10 min 100/0%, 11 min 5/95%, and 12 min 5/95% at a flow rate of 0.4 mL/min. The proportion of the organic mobile phase between 2 and 8 min was increased non-linearly by a convex upward curve no. 2 available in Chromeleon. The electrospray ion source dry temperature was set to 250°C, drying gas flow to 11 L/min, and nebulizer pressure to three bars. Capillary voltage was set to 4,500 V and endplate offset to 500 V. The spectra were collected in the range of 50 to 1,490 m/z with a spectral rate of 2 Hz. Precursor ions were selected automatically, and collision-induced dissociation (CID) was set as a ramp from 20 to 50 eV. Calibration of the instrument was performed using CH_3_COONa clusters at the beginning of each analysis. Data were analyzed using Bruker Data Analysis software and MZmine 3 (42).

### Isolation of cyanochelin B

Spent media of iron-deprived culture of *Leptolyngbya* with HPLC-HRMS-confirmed content of cyanochelin B was shaken overnight with amberlite XAD-16, then rinsed with 10% MeOH (≥99.9%, gradient grade for HPLC, VWR Chemicals BDH, Avantor, cat. no. 83638.320) and eluted with 100% MeOH. The extracts were evaporated on a rotary evaporator (70 mBar, 40°C) and stored in the freezer. Before HPLC separation, the extracts were dissolved in a minimal volume of MeOH. The compounds were isolated on a µBONDAPAK phenyl column (7.8mm × 30 mm, Waters) using acetonitrile (ACN, solvent A) and 25% vol/vol acetonitrile-water mixture (solvent B). The solvents were amended with trifluoroacetic acid (TFA, 0.002% and 0.08% vol/vol, respectively), which proved to be crucial for preventing unspecific compound-column interactions and ensuring reliable elution. The column was eluted with the following linear gradient: 0 min, 100% B, 3 mL/min; 2 min, 100% B, 3 mL/min; 4 min, 100% B, 4 mL/min; 8 min, 50% B, 4 mL/min; 24 min 65% B, 4 mL/min; 25 min, 100% B, 4 mL/min; 28 min, 100% B, 4 mL/min; 29 min, 0%B, 3 mL/min; 30 min, 0% B, 3 mL/min. Column temperature was maintained at 40°C. Fractions were collected by an automated fraction collector on a time basis with 0.75 min windows starting from minute 2 and ending at minute 27. Collected fractions were checked for the content of the compound of interest by HPLC-HRMS (see above), pooled accordingly, and dried. If needed, pooled prepurified extracts with high 1026-content were subjected to a second round of HPLC isolation on the same column with an analogous MeOH/water gradient. The obtained fractions were selected based on the data and evaporated.

### Structural characterization of cyanochelin B

^1^H NMR and 2D NMR experiments were carried out at 35°C on a Bruker AvanceNeo 700 MHz spectrometer (Billerica, MA, USA) equipped with a triple resonance CHN cryoprobe, using d-DMSO (Sigma Aldrich, Milan, Italy). Chemical shifts were referenced to the residual solvent signal (d-DMSO: δ_H_ 2.50 ppm, δ_C_ 39.51 ppm). The heteronuclear single quantum correlation (HSQC) spectra were optimized for ^1^*J*_CH_ = 145 Hz. The HMBC experiments were optimized for ^2,3^*J*_CH_ = 8 Hz. Abbreviations for signal couplings are as follows: s = singlet, d = doublet, br.d = broad doublet, dd = doublet of doublets, td = triplet of doublets, t = triplet, q = quartet, m = multiplet.

### Advanced Marfey’s analysis

The cyanochelin B (10 µg) was hydrolyzed with 600 µL of 6N HCl/AcOH (1:1) at 120°C for 18 h. The residual HCl fumes were removed under an N_2_ stream. The hydrolysate was then dissolved in triethylamine (TEA)/acetone (2:3, 200 µL), and the solution was treated with 200 µL of 1% 1-fluoro-2,4-dinitrophenyl-5-l-alaninamide (l-FDAA) in CH_3_CN/acetone (1:2) (43). Subsequently, another aliquot of cyanochelin B (10 µg) was hydrolyzed, as above, and derivatized with 200 µL of 1% 1-fluoro-2,4-dinitrophenyl-5-d-alaninamide (d-FDAA). The vial was heated at 50°C for 2 h. The mixture was dried, and the resulting l-DAA and d-DAA derivatives of the free amino acids were redissolved in MeOH (200 µL) for subsequent analysis. Authentic standards of l-Tyr, d-Ser, and l-β-OH-Asp were treated with l-FDAA and d-FDAA as described above and yielded the l-DAA and d-DAA standards. Marfey’s derivatives of cyanochelin B were analyzed by using the high-performance liquid chromatography system (Thermo U3000 HPLC system) connected to a Thermo LTQ Orbitrap XL mass spectrometer (Thermo Fisher Scientific Inc., Waltham, MA, USA), and their retention times were compared with those from the authentic standard derivatives. A 5 µm Kinetex C18 column (50 × 2.10 mm) maintained at 25°C was eluted at 200 µL min^–1^ with 0.1% HCOOH in H_2_O and ACN. The gradient program was as follows: 5% ACN, 3 min; 5%–50% ACN over 30 min; 50%–90% ACN over 1 min; and 90% ACN, 6 min.

Mass spectra were acquired in positive ion detection mode. MS parameters utilized a spray voltage of 4.8 kV, a capillary temperature of 285°C, a sheath gas rate of 32 units N_2_ (ca. 230 mL/min), and an auxiliary gas rate of 15 units N_2_ (ca. 150 mL/min). The MS method involved four HRMS/MS scans after each full MS scan for the four most intense ions detected in the spectrum (data-dependent acquisition mode, DDA). The m/z range for data-dependent acquisition was set between 150 and 2,000 amu, with resolution set to 60,000. HRMS/MS scans were obtained for selected ions with CID fragmentation using an isolation width of 4.0, a normalized collision energy of 35, an activation Q of 0.250, and an activation time of 30 ms. Mass data were analyzed using the Xcalibur suite of programs.

Analysis of mass spectra of both β-OH-Asp standards and the cyanochelin-l-DAA and cyanochelin-d-DAA was carried out by using a 3 µm Phenomenex Luna Omega Polar C18 (100 × 2.10 mm) maintained at 45°C and eluted at 400 µL min^–1^ with 0.1% HCOOH in H_2_O and ACN. The gradient program was as follows: 10% ACN, 1.5 min; 10%–95% ACN over 8.5 min; and 95% ACN, 2 min.

The analysis indicated that the amino acid forms various adducts with the derivatizing agent, prompting further analysis of chromatograms from LC-MS SRM (selected reaction monitoring) spectra. In SRM, mass analyzers are set to a selected mass-to-charge ratio, focusing on specific precursor and product ions. The MS method involved two HRMS/MS scan events, following two product ions: m/z 356.1–358.1 and m/z 280.1–282.1 from the m/z precursor 402.2.

### Determination of kinetics of cyanochelin B photolysis

Cyanochelin B (10 µM) was mixed with excess FeCl_3_ (20 µM) in 10 mM ammonium acetate buffer (pH = 5) and incubated overnight at room temperature to ensure formation of iron-siderophore complexes. The buffer was chosen for its compatibility with MS. A control mixture without addition of FeCl_3_ was prepared analogously. Aliquots of the solution (75 µL) were distributed to individual round-bottom wells of a 96-well plate (VWR art. no. 734-2803). The plate was placed under the source of UV-A and visible light provided by LED strips, or outside to natural sunlight. The intensity of visible light was constant in all indoor variants of the experiment (50 µmol m^−2^ s^−1^). Four intensities of UV light were used: UV0: UV-A light source was off; UV1: UV-A 1.4 µmol m^−2^ s^−1^; UV2: UV-A 2.5 µmol m^−2^ s^−1^; UV3: UV-A 4.1 µmol m^−2^ s^−1^; UV4: UV-A light source set to 2.0 µmol m^−2^ s^−1^ and the well plate was covered with the lid as during cultivation of cyanobacteria; and UV5: exposed to natural sunlight at intensity of UV-A 19.5 µmol m^−2^ s^−1^. At given time points, the timer was stopped, the plate was removed from the source of UV light, and 75 µL of MeOH was added to the well. The content of the well was mixed thoroughly, and the solution was transferred to a glass vial. The plate was returned to designated light conditions, and the timer was resumed. The sample was protected from light, and the sample was analyzed by HPLC-HRMS within 24 h. The data were processed using MZmine 3 to quantify the content of cyanochelin B. Linear regression was applied on the log-transformed integrated peak areas of cyanochelin B to confirm the order of the reaction and determine its rate.

### Field sampling and strain isolation

A microbial mat growing on a splashed surface of a wooden bridge over a stream near a waterfall at GPS coordinates N 44.04152, E O16.23488, altitude 87 m, was collected as sample 146 by scraping the surface with an ethanol-cleaned spatula and placed in a sterile 50 mL tube. Later the same day, the sample was examined by an Olympus CX23 light microscope equipped with an oil-less 60× objective. After confirmation that the mat was dominated by cyanobacteria, the sample was (i) split into disposable plastic cultivation bottles in iron-deprived and standard Z medium (44); (ii) placed in a sterile cryo-vial in a travel container with liquid nitrogen to preserve the original community for DNA analysis; (iii) smashed and preserved with 20% glycerol and stored as a backup. The sample was then kept in a travel fridge (~8°C) with an LED light to transfer the live community. Once the multispecies culture derived from sample 146 was confirmed to produce cyanochelin B by HPLC-MS analysis, it was inspected by optical microscope, and single filaments of the dominant morphotypes were picked by a sterile glass capillary and inoculated into fresh Z medium to obtain clonal cultures (45).

### Cyanochelin B PCR protocol

Based on sequences of the previously identified cyanochelin B BGC of *Leptolyngbya* sp. NIES-3755 (12) and a homologous BGC harbored in the genome of *Myxacorys almedinensis* A (ULC467—GCA_010091945.1) disclosed by the BLAST search, a PCR protocol amplifying five loci scattered among the cluster was designed (Table S6). Whole genomic DNA from sample 146 and from *Leptolyngbya* sp. NIES-3755 (applied as a positive control) was isolated using NucleoSpin Soil Mini kit (Macherey Nagel, Düren, Germany) following the manufacturer’s instructions. The PCR amplification was performed using each loci-specific primer pairs and sent for Sanger sequencing (SeqME, Dobříš, Czech Republic) as described previously (46). The protocol confirmed the occurrence of cyanochelin B BGC in the mixed sample 146, being positive in three loci (1, 4, and 5). Moreover, the occurring single-nucleotide polymorphisms indicated cluster variants. The designed protocol was further used to screen isolated clonal strains to identify potential producers. DNA from three strains of each *Leptolyngbya*-like morphotype was isolated (Table S4), PCR executed, and positive loci prepared for sequencing (as described previously). Taxonomic identity of three strains of all four morphotypes was confirmed by 16S rRNA PCR amplification and sequencing using VRF2 (5´– GGG GAA TTT TCC GCA ATG GG –3´) and VRF1 (5´– CTC TGT GTG CCT AGG TAT CC –3´) primers and PCR setting described in Johansen et al. (47).

### Whole genome sequencing and assembly

Single filaments of each of the five clonal strains of *Phormidesmis* sp. from sample 146 were isolated by glass capillary technique, and the genomic DNA was amplified using multiple-displacement amplification (MDA) with the Repli-G Mini Kit (Qiagen, Hilden, Germany) as described previously (48). MDA products from five to eight filaments per strain that passed a quality check by 16S rRNA sequencing were pooled together and sent for *de novo* genome sequencing (Institute of Experimental Botany, Center of Plant Structural and Functional Genomics, Olomouc, Czech Republic) using an Illumina HiSeq Pair-End library with 150 bp reads and 2.5 Gbp data yield per sample. The raw data from Illumina were trimmed, assembled, binned to remove minor bacterial contamination, and taxonomically classified using the *nf-core/mag* metagenomics pipeline (49) installed on the server of the Institute of Hydrobiology, Biology Centre of the CAS.

### Phylogenetic analysis

The final rRNA operon data of *Phormidesmis* sp. SID-146-12, 20, 33, and 35 were excised from their sequenced genomes (Geneious Prime 2024.0.7); for *Phormidesmis* sp. SID-146-36, only a partial sequence was obtained from a Sanger-sequenced PCR product amplified by the primers VRF2 (5´– GGG GAA TTT TCC GCA ATG GG –3´) and 1494 (5´– CTA CGG CTA CCT TGT TAC GA –3´) (47, 50). Data were deposited in the NCBI GenBank database under the accession numbers PQ675769–PQ675773. Obtained sequences were aligned with a wide range of *Leptolyngbyaceae* representatives, including all major lineages inside the family. The final matrix consisted of 108 taxa, including 3 taxa of *Oculatellaceae* employed as an outgroup, and it was 1,112 positions long. The alignment was constructed using MEGA11 (51) by the MUSCLE (52) algorithm and adjusted manually. Bayesian inference was executed in the program MrBayes v.2.6 (53) with default settings for 5 million generations. Posterior probabilities of data split were recorded for individual branches. Maximum likelihood analysis was performed in the PhyML software (54) applying the best model TN93+R automatically chosen by SMS algorithm (55). Nonparametric bootstrap of 1,000 pseudo-replications was calculated and used as branch support. Obtained trees were merged, and the final figure was adjusted in graphic illustrator InkScape (https://inkscape.org/). Only supports above 50 (ML) and above 0.95 (posterior probability) are displayed. For novel strains, the check of p-distances of the rRNA as well as cyanochelin B BGC similarity was done using MEGA11.

### Analysis of the cyanochelin BGCs

The genomic bins of *Phormidesmis* sp. strains were automatically screened for secondary metabolite BGCs using the antiSMASH v.7.0 online analysis tool in the bacterial version (56). Contigs containing tentative BGCs with similarity to cyanochelin A in KnownClusterBlast were analyzed in detail to address the composition of NRPS and PKS domains, the predicted substrate specificity of amino acid adenylation domains (Stachelhaus code), the presence of amino acid epimerase domains, and the identity of additional tailoring enzymes to aid in cyanochelin structure elucidation and to link the BGC to its product. Stereoconfiguration of the PKS ketoreductase (KR) domain substrate was predicted based on an alignment of bacterial KR domains of various types together with the KR domain encoded in the *ccsAB* gene, using an analysis of conserved residues within the active site groove (26).

## Data Availability

The relevant data generated from this study are publicly available. Genome sequences were deposited at NCBI GenBank under accession numbers JBJPHS000000000, JBJPHT000000000, JBJPHU000000000, and JBJPHV000000000. The NMR data used for structure elucidation of cyanochelin B have been deposited in the Natural Products Magnetic Resonance Database (NP-MRD; www.np-mrd.org) and can be found under accession number NP0351369. The HRMS data regarding the photolysis of the compound can be provided on request by the corresponding author.

## SUPPLEMENTAL MATERIAL

Supplemental material aem.02566-24-s0001.pdf Tables S1 to S6; Fig. S1 to S21.

ASM does not own the copyrights to Supplemental Material that may be linked to, or accessed through, an article. The authors have granted ASM a non-exclusive, world-wide license to publish the Supplemental Material files. Please contact the corresponding author directly for reuse.
