# Supplementary material for: Cyanochelin B: a cyanobacterium-produced siderophore with photolytic properties that negate iron monopolization in UV light
Source: Appl Environ Microbiol. 2025 Oct 15;91(11):e02566-24. doi: 10.1128/aem.02566-24 (PMC12628792; doi:10.1128/aem.02566-24)
Supplement: Supplemental material — Tables S1 to S6; Fig. S1 to S21. [file aem.02566-24-s0001.pdf]

# Supplementary material to article:

# Cyanochelin B: A cyanobacterium-produced siderophore with photolytic properties that negate iron monopolization in the UV-light

Berness P. Falcao[1ab], Viviana Di Matteo[1c], Pavel Hrouzek[ab], Lenka Štenclová[de], Petra Urajová[a], Jan Mareš[ad], Jan Kuta[e], José Alberto Martínez Yerena[abd], Eliška Kozlíková[d], Germana Esposito[c], Alfonso Mangoni[c], Valeria Costantino[c]*, Tomáš Galica[a]*

[1] - authors contributed equally

* - corresponding authors

a - Centre Algatech, Institute of Microbiology of the Czech Academy of Sciences, Třeboň 37901, Czech Republic

b - Faculty of Science, University of South Bohemia, České Budějovice 37005, Czech Republic

c - The Blue Chemistry Lab Group, Department of Pharmacy, Università degli Studi di Napoli Federico II, 80131 Napoli, Italy

d - Biology Centre of the Czech Academy of Sciences, Institute of Hydrobiology, České Budějovice 37005, Czech Republic; c

e - Department of Pharmaceutical Biology, Institute of Pharmacy, Freie Universität Berlin, 14195 Berlin, Germany

f - RECETOX, Faculty of Science, Masaryk University, Kotlarska 2, Brno, Czech Republic

**Table S1: Annotation of ions found in MS2 spectra of cyanochelin B**

| Ion | m/z | Molecular Formula | Error (ppm) |
|---|---|---|---|
| $[M+H]^+$ | 1026.5065 | $C_{47}H_{76}N_7O_{16}S^+$ | 0.1 |
| $[M\text{-FA}+H]^+$ | 786.2613 | $C_{31}H_{44}N_7O_{15}S^+$ | 0.3 |
| $[M\text{-FA-ethanolamine}+H]^+$ | 725.2580 | $C_{29}H_{37}N_6O_{14}S^+$ | 0.5 |
| [M-FA-ethanolamine-*β*-OH-Asp+H]$^+$ | 594.1889 | $C_{25}H_{32}N_5O_{10}S^+$ | 0.1 |
| [M-FA-ethanolamine-*β*-OH-Asp-Ser+H]$^+$ | 507.1543 | $C_{22}H_{27}N_4O_8S^+$ | 1.4 |
| [M-FA-ethanolamine-*β*-OH-Asp-Ser-*β*-OH-Asp+H]$^+$ | 376.1325 | $C_{18}H_{22}N_3O_4S^+$ | 0.2 |
| [M-FA-ethanolamine-*β*-OH-Asp-Ser-*β*-OH-Asp-Gly+H]$^+$ | 319.1107 | $C_{16}H_{19}N_2O_3S^+$ | 1.1 |
| [M-FA-ethanolamine-*β*-OH-Asp-Ser-*β*-OH-Asp-Gly-CO+H]$^+$ | 291.1160 | $C_{15}H_{19}N_2O_2S^+$ | 0.6 |
| [M-FA-ethanolamine-*β*-OH-Asp-Ser-*β*-OH-Asp-Gly-CO-Phe+H]$^+$ | 144.0474 | $C_6H_{10}NOS^+$ | 2.2 |

**Table S2:** Specificities of the A-domains in the identified biosynthetic gene clusters. A-domains in the order of synthesis are coded as E, F, G, I, J, L and M

| Strain | A-domain | Protein | Position | Stachelhaus Prediction | Stachelhaus Code | Reconstructed Substrate | Observed AA |
|---|---|---|---|---|---|---|---|
| *M. almedinensis A* | A1 | CcsE | 1407-1803 | Cys | DLYNLSLIWK | Cys | Cys |
| | A2 | CcsFGH | 505-896 | Phe | DAWTIAAVCK | Phe | Phe |
| | A3 | | 1551-1942 | Gly | DILQLGLIWK | Gly | *Gly* |
| | A4 | CcsI | 450-850 | Asp | DLTKIGHVGK | Asp | *ß*-OH-Asp |
| | A5 | CcsJ | 510-911 | Ser | DVWHFSLIDK | Ser | Ser |
| | A6 | CcsL | 450-853 | Asp | DLTKIGHVGK | Asp | *ß*-OH-Asp |
| | A7 | CcsM | 470-863 | Gly | DILQLGLIWK | Gly | Ethanolamine |
| *Phormidesmis* sp. 146-12 | A1 | CcsE | 1405-1802 | Cys | DLYNLSLIWK | Cys | Cys |
| | A2 | CcsFGH | 504-895 | Phe | DAWTIAAVCK | Phe | Phe |
| | A3 | | 1550-1941 | Gly | DILQLGLIWK | Gly | *Gly* |
| | A4 | CcsI | 448-848 | Asp | DLTKVGHVGK | Asp | *ß*-OH-Asp |
| | A5 | CcsJ | 510-911 | Ser | DVWHFSLIDK | Ser | Ser |
| | A6 | CcsL | 451-854 | Asp | DLTKIGHVGK | Asp | *ß*-OH-Asp |
| | A7 | CcsM | 470-862 | Gly | DILQLGLIWK | Gly | Ethanolamine |
| *Phormidesmis* sp. 146-20 | A1 | CcsE | 1406-1802 | Cys | DLYNLSLIWK | Cys | Cys |
| | A2 | CcsFGH | 504-895 | Phe | DAWTIAAVCK | Phe | Phe |
| | A3 | | 1550-1941 | Gly | DILQLGLIWK | Gly | *Gly* |
| | A4 | CcsI | 448-848 | Asp | DLTKVGHVGK | Asp | *ß*-OH-Asp |
| | A5 | CcsJ | 510-911 | Ser | DVWHFSLIDK | Ser | Ser |
| | A6 | CcsL | 452-855 | Asp | DLTKIGHVGK | Asp | *ß*-OH-Asp |
| | A7 | CcsM | 470-862 | Gly | DILQLGLIWK | Gly | Ethanolamine |
| *Phormidesmis* sp. 146-33 | A1 | CcsE | 1406-1802 | Cys | DLYNLSLIWK | Cys | Cys |
| | A2 | CcsFGH | 504-895 | Phe | DAWTIAAVCK | Phe | Phe |
| | A3 | | 1550-1941 | Gly | DILQLGLIWK | Gly | *Gly* |
| | A4 | CcsI | 449-849 | Asp | DLTKVGHVGK | Asp | *ß*-OH-Asp |
| | A5 | CcsJ | 510-911 | Ser | DVWHFSLIDK | Ser | Ser |
| | A6 | CcsL | 452-855 | Asp | DLTKIGHVGK | Asp | *ß*-OH-Asp |
| | A7 | CcsM | 470-862 | Gly | DILQLGLIWK | Gly | Ethanolamine |
| *Phormidesmis* sp. 146-35 | A1 | CcsE | 1406-1802 | Cys | DLYNLSLIWK | Cys | Cys |
| | A2 | CcsFGH | 504-895 | Phe | DAWTIAAVCK | Phe | Phe |
| | A3 | | 1550-1941 | Gly | DILQLGLIWK | Gly | *Gly* |
| | A4 | CcsI | 448-848 | Asp | DLTKVGHVGK | Asp | *ß*-OH-Asp |
| | A5 | CcsJ | 510-911 | Ser | DVWHFSLIDK | Ser | Ser |
| | A6 | CcsL | 452-855 | Asp | DLTKIGHVGK | Asp | *ß*-OH-Asp |
| | A7 | CcsM | 470-862 | Gly | DILQLGLIWK | Gly | Ethanolamine |

**Table S3: Summary of NMR analyses of cyanochelin B.** (1H 700 MHz, 13C 175 MHz, DMSO-d6, 308K)

| Residue | Position | | δH [mult., J (Hz)] | δC [mult.] | HMBC ($^1$H→$^{13}$C) | NOESY |
|---|---|---|---|---|---|---|
| | OH-1 | | | | | |
| | 1 | | 3.37, t (6.5) | 59.6, $CH_2$ | 2 | 2, NH-2 |
| | 2 | | 3.13, m | 41.8, $CH_2$ | 1, 3 | 1, NH-2 |
| | NH-2 | | 7.68, t (5.6) | | 1, 2, 3 | 1,2, NH-4 |
| D-β-OH-Asp | 3 | | | 168.8, C | | |
| | 4 | | 4.69, dd (9.1, 2.3) | 56.0, CH | 3, 6, 7 | NH-2, NH-4 |
| | 5 | | 4.60, d (2.3) | 69.7, CH | 3, 6, 7 | NH-2, NH-4 |
| | OH-5 | | | | | |
| | 6 | | | 173.3, C | | |
| | NH-4 | | 7.93, d (9.1) | | 4, 7 | NH-2, NH-8, NH-11 |
| D-Ser | 7 | | | 170.2, C | | |
| | 8 | | 4.45, td (7.4, 5.8) | 54.5, CH | 7, 10 | NH-4, NH-8 |
| | 9 | a | 3.67, dd (5.8, 10.2) | 62.0, CH | 7 | NH-4, NH-8 |
| | | b | 3.47, dd (7.3 ,10.2) | | 7 | NH-4, NH-8 |
| | OH-9 | | | | | |
| | NH-8 | | 7.83, d (7.5) | | 8, 10 | NH-4, NH-11,11 |
| L-β-OH-Asp | 10 | | | 169.0, C | | |
| | 11 | | 4.76, dd (9.1, 2.7) | 55.4, CH | 10,13,14 | NH-8, NH-11 |
| | 12 | | 4.51, d (2.7) | 70.2, CH | 10, 13,14 | NH-8, NH-11 |
| | OH-12 | | | | | |
| | 13 | | | 172.9, C | | |
| | NH-11 | | 8.07, d (9.1) | | 10,11, 14 | NH-8, NH-15 |
| Gly | 14 | | | 169.0, C | | |
| | 15 | a | 3.89, dd (17.0, 5.6) | 41.8, $CH_2$ | 14, 16 | NH-15, NH-11 |
| | | b | 3.80, dd (17.0, 5.6) | | 14, 16 | NH-15, NH-11 |
| | NH-15 | | 8.27, t (5.6) | | 15, 16 | NH-11, NH-17, 28 |
| L-Phe | 16 | | | 171.0, C | | |
| | 17 | | 4.60, td (9.5, 4,4) | 53.6, CH | 16, 19,25 | NH-15, NH-17 |
| | 18 | a | 3.05, dd (13.6, 4.4) | 38.0, $CH_2$ | 16,17, 19 | NH-15, NH-17 |
| | | b | 2.79, dd (13.6, 10.1) | | 16,17,19 | NH-15, NH-17 |
| | 19 | | | 137.6, C | | |
| | 20/24 | | 7.20, m | 129.3, CH | 18, 19 | 17 |
| | 21/23 | | 7.20, m | 127.8, CH | 18, 19 | |
| | 22 | | 7.16, m | 126.1, CH | | |
| | NH-17 | | 7.65, d (9.0) | | 17, 25 | NH-15, 28 |
| | 25 | | | 173.5, C | | |
| | 26 | | | 84.0, C | | |
| | 27 | a | 2.97, d (11.3) | 40.7, $CH_2$ | 25, 26, 28, 29 | 28 |
| | | b | 2.82, d (11.3) | | 25, 26, 28, 29 | 28 |
| | 28 | | 1.25, s | 23.8, CH3 | 25, 26, 27 | 27a ,27b |
| | 29 | | | 178.7, C | | |
| | 30 | | | 76.8, C | | |
| | OH-30 | | | | | |
| | 31 | | 1.34, s | 24.0, $CH_3$ | 29, 30, 32 | 33a, 33b, 32 |
| | 32 | | 3.47, br.d (9.9) | 76.0, $CH_2$ | 29,34 | 31 |
| | OH-32 | | | | | |
| | 33 | a | 1.51, m | 30.5, $CH_2$ | 34,35/43 | 31,32 |
| | | b | 1.23, m | | 34,35/43 | |
| | 34 | a | 1.50, m | 26.2, $CH_2$ | 32,33, 35/45, 44 | 32,35/43 |
| | | b | 1.25, m | | 32,33, 35/45, 44 | |
| | 35/43 | | 1.23, m | 29.0, $CH_2$ | 34,44 | 34a,34b |
| | 44 | | 1.24, m | 28.6, $CH_2$ | 35/43,45,46 | |
| | 45 | | 1.24, m | 31.3, $CH_2$ | 35/43,44,46 | |
| | 46 | | 1.26, m | 22.1, $CH_2$ | 44, 45, 47 | 47 |
| | 47 | | 0.86, t (7.1) | 13.9, $CH_3$ | 45, 46 | 46 |

**Table S4:** Comparison of UV-A (315 - 400 nm) and visible light (VIS, 400 - 800 nm) intensities used in photolysis and cultivation experiments as well as representative measurements of natural light. SpectraPen (Photon System Instruments) was used to do the measurements and for quick readout of the intensities. The measured spectra were exported and processed using python3 pandas library to integrate and quantify the intensities of light in the given ranges.

| | Photon Flux density [μmol/m²/s] | | Irradiance [W/m²] | | | |
|---|---|---|---|---|---|---|
| | UV-A (315-400 nm) | VIS (400-800nm) | UV-A (315-400 nm) | VIS (400-800nm) | Measurement date | Measurement no. |
| UV0 (noUV) | 0.12 | 45.29 | 0.04 | 9.65 | 2023-09-21 | 1 |
| UV1 (min UV) | 1.41 | 45.86 | 0.46 | 9.77 | 2023-09-21 | 2 |
| UV2 (½ UV) | 2.53 | 46.20 | 0.82 | 9.84 | 2023-09-21 | 3 |
| UV3 (max UV) | 4.14 | 47.04 | 1.34 | 10.03 | 2023-09-21 | 4 |
| UV4 (½ UV + lid) | 2.06 | 47.19 | 0.67 | 10.06 | 2023-09-21 | 5 |
| UV0 (noUV, 2$^{nd}$ photolysis ) | 0.20 | 54.31 | 0.07 | 11.57 | 2025-03-24 | 7 |
| UV2 (½ UV, 2$^{nd}$ photolysis) | 2.75 | 48.84 | 0.89 | 10.40 | 2025-03-24 | 11 |
| OUT1 (noon, no cloud) | 29.53 | 1468.51 | 9.58 | 294.5 | 2025-03-24 | 1 |
| OUT2 (~2pm, partly cloudy) | 14.23 | 653.69 | 4.61 | 132.12 | 2025-03-24 | 14 |
| OUT3 (~2pm, partly cloudy) | 12.04 | 478.56 | 3.90 | 97.43 | 2025-03-24 | 18 |

**Table S5:** Overview of the cyanobacterial strains isolated from the field sample no. 146, investigated in present study.

| Strain | Morphotype | Taxon | PCR+ *loci* | 16S rRNA | ITS | Genome | Cluster | Production |
|---|---|---|---|---|---|---|---|---|
| SID-146-02 | Bright green, hairy colony | Leptolyngbyaceae sp. 1 | | Y | 2 variants | | | |
| SID-146-09 | Bright green, hairy colony | Leptolyngbyaceae sp. 1 | | Y | 2 variants | | | |
| SID-146-10 | Bright green, hairy colony | Leptolyngbyaceae sp. 1 | | Y | 2 variants | | | |
| SID-146-12 | Cyan-green short cells | *Phormidesmis* sp. | 4, 5 | Y | Y | Y | Y | Y |
| SID-146-15 | Cyan-green thin long cels | *Agnostidinema* | | Y | Y | | | |
| SID-146-18 | Black | Leptolyngbyaceae sp. 2 | | Y | Y | | | |
| SID-146-20 | Cyan-green short cells | *Phormidesmis* sp. | 1, 4, 5 | Y | Y | Y | Y | N |
| SID-146-23 | Cyan-green thin long cels | *Agnostidinema* | | Y | Y | | | |
| SID-146-24 | Black | Leptolyngbyaceae sp. 2 | | Y | Y | | | |
| SID-146-32 | Black | Leptolyngbyaceae sp. 2 | | Y | Y | | | |
| SID-146-33 | Cyan-green short cells | *Phormidesmis* sp. | 4, 5 | Y | Y | Y | Y | Y |
| SID-146-34 | Cyan-green thin long cels | *Agnostidinema* | | Y | Y | | | |
| SID-146-35 | Cyan-green short cells | *Phormidesmis* sp. | N/A | Y | Y | Y | Y | Y |
| SID-146-36 | Cyan-green short cells | *Phormidesmis* sp. | N/A | partial | N | partial | N | Y |

**Table S6:** Primers used for the PCR screening for cyanochelin B biosynthetic gene cluster

| ORF | Genes | Primer - Position (Bp) | Sequence |
|---|---|---|---|
| **ORF1** | **A/B** | | |
| **ORF2** | **C** | | |
| **ORF4** | **D/E** | **CCH_B_1 - 12,970 F** | **TATGAAATTCACGAGTGGGA** |
| | | **CCH_B_1 - 13,591 R** | **GATCATTGACTTGAGGATGC** |
| | | CCH_B_2 - 13,530 F | TCACTTCACGATTAACCTGA |
| | | CCH_B_2 - 14,097 R | ATTGAGGGTCGTCTGTAAAT |
| **ORF5** | **FGH** | CCH_B_3 - 23,727 F | CCATTGTTGATCTCAGTAGC |
| | | CCH_B_3 - 24,373 R | GATCACCAGCAAATTAACGA |
| **ORF7** | **I** | | |
| **ORF8** | **J** | **CCH_B_4 - 34,547 F** | **AGGATGTAGTATTTGGTGCA** |
| **ORF9** | **K** | **CCH_B_4 - 35,145 R** | **TCGCTTAGTCAATTTCAACC** |
| | | **CCH_B_5 - 35,829 F** | **GGGACAGGATCAGTTAAGAA** |
| **ORF10** | **L** | **CCH_B_5 - 36,462 R** | **CATCTCTGGCAAACATTGAA** |
| **ORF12** | **M** | | |

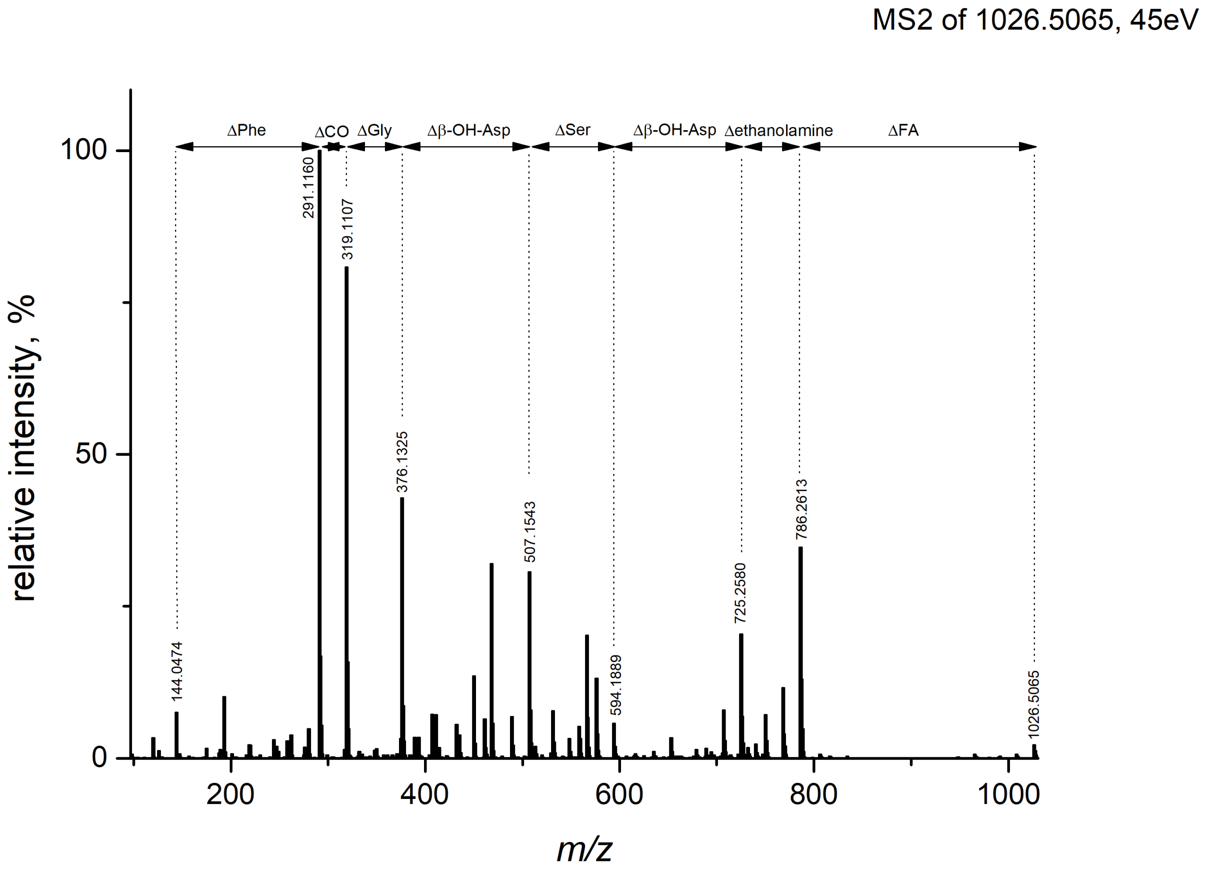


**Fig. S1: Annotated MS/MS fragmentation spectrum of cyanochelin B (1026.5065)**

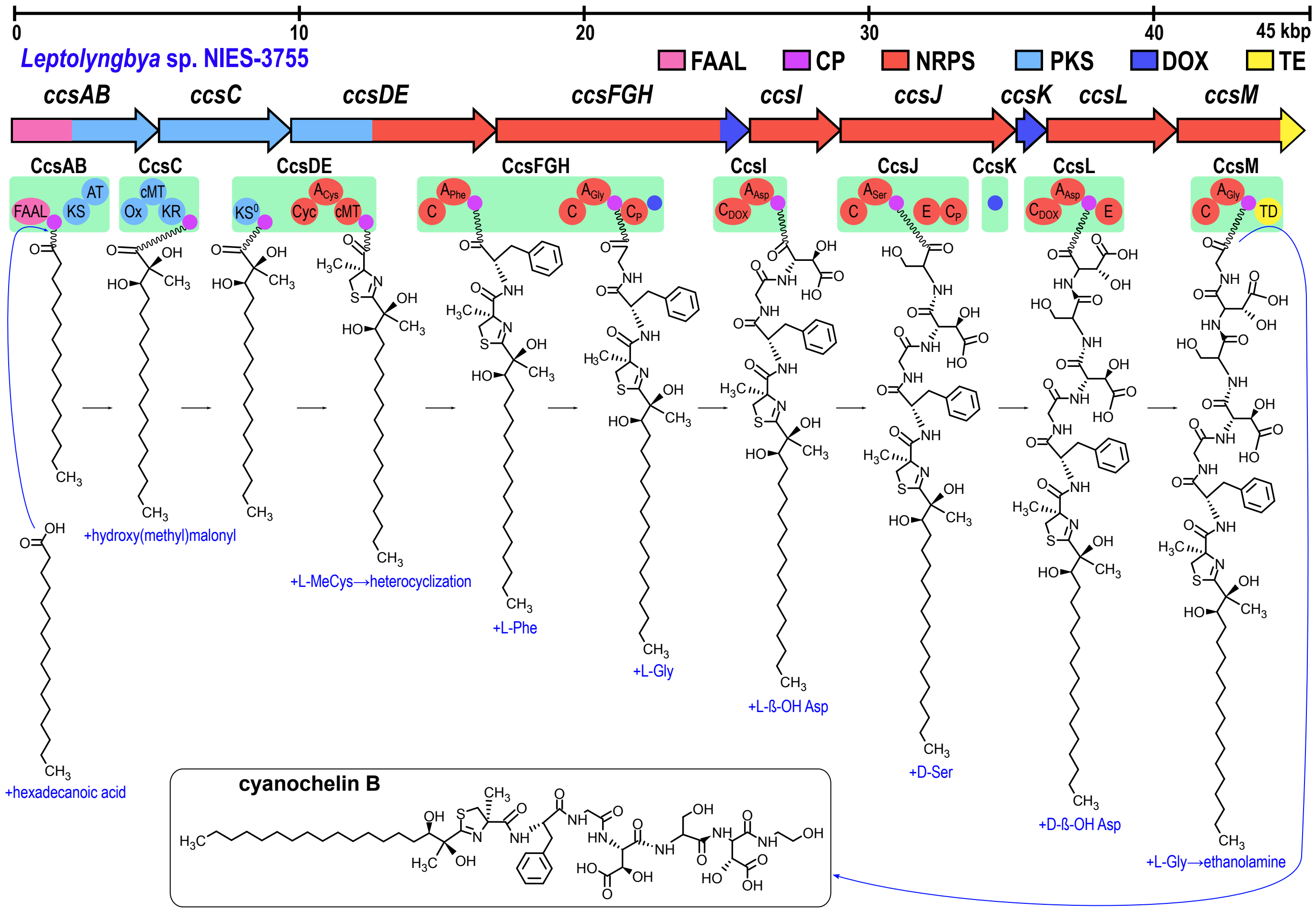


**Fig. S2: Reconstruction of cyanochelin B biosynthesis in *Leptolyngbya* sp. NIES-3755.** Cyanochelin B is synthesized by a PKS/NRPS pathway enconded by 9 nine genes grouped in ~45 kbp long BGC depicted by arrows. Genes genes are depicted in scale and portion of the genes are colorized by according to type of biosynthetic step they perform. Multidomain proteins (green frames) are depicted with individual domains and attached intermediate. Abbreviations: FAAL - fatty acyl-AMP ligase, CP - carrier protein, NRPS - non-ribosomal peptide synthetase, PKS - polyketide synthetase, DOX - aspartate $\beta$-hydroxylase, TE - thiesterase

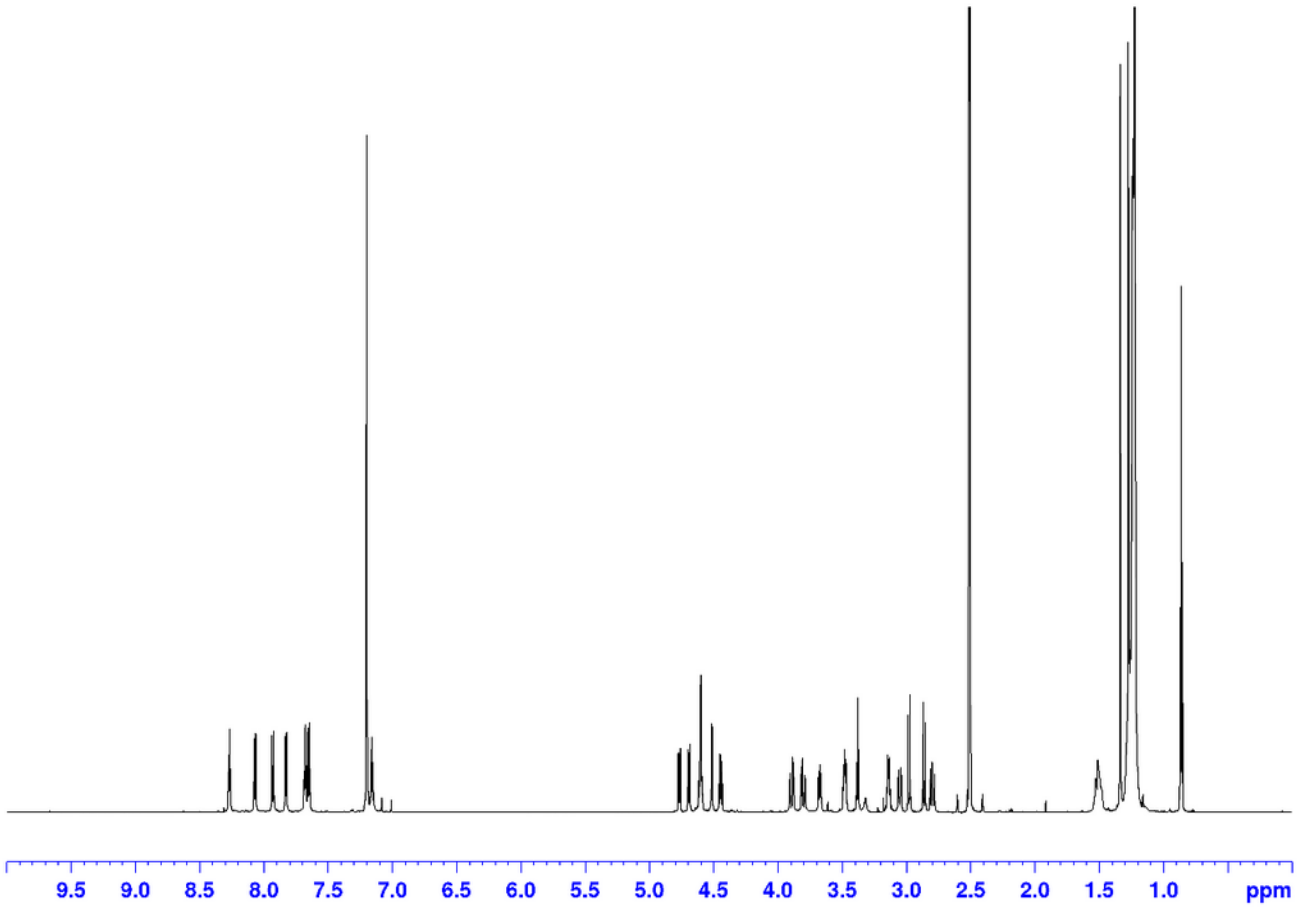


**Fig. S3:** $^{1}$H-NMR spectrum of cyanochelin B (700 MHz, DMSO-d6, 308K)

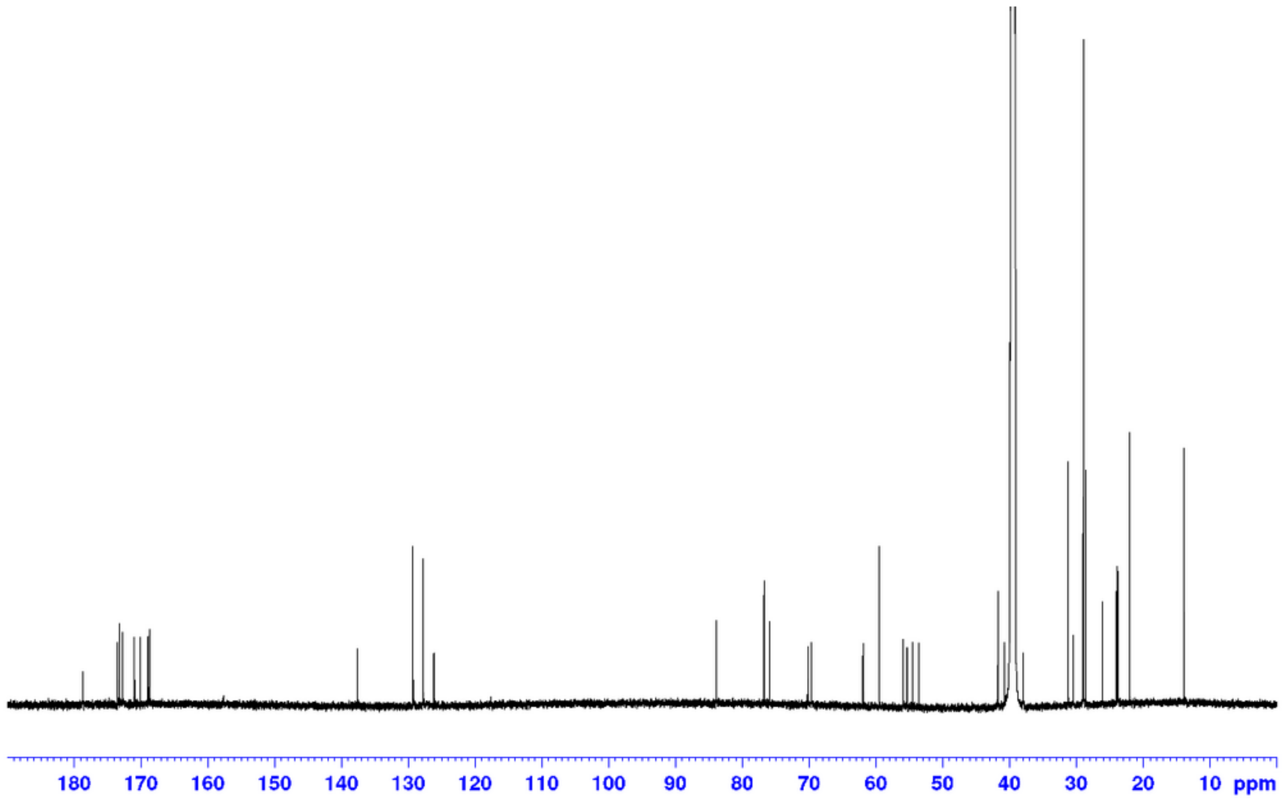


**Fig. S4:** $^{13}C$-NMR spectrum of cyanochelin B (700 MHz, DMSO-d6, 308K)

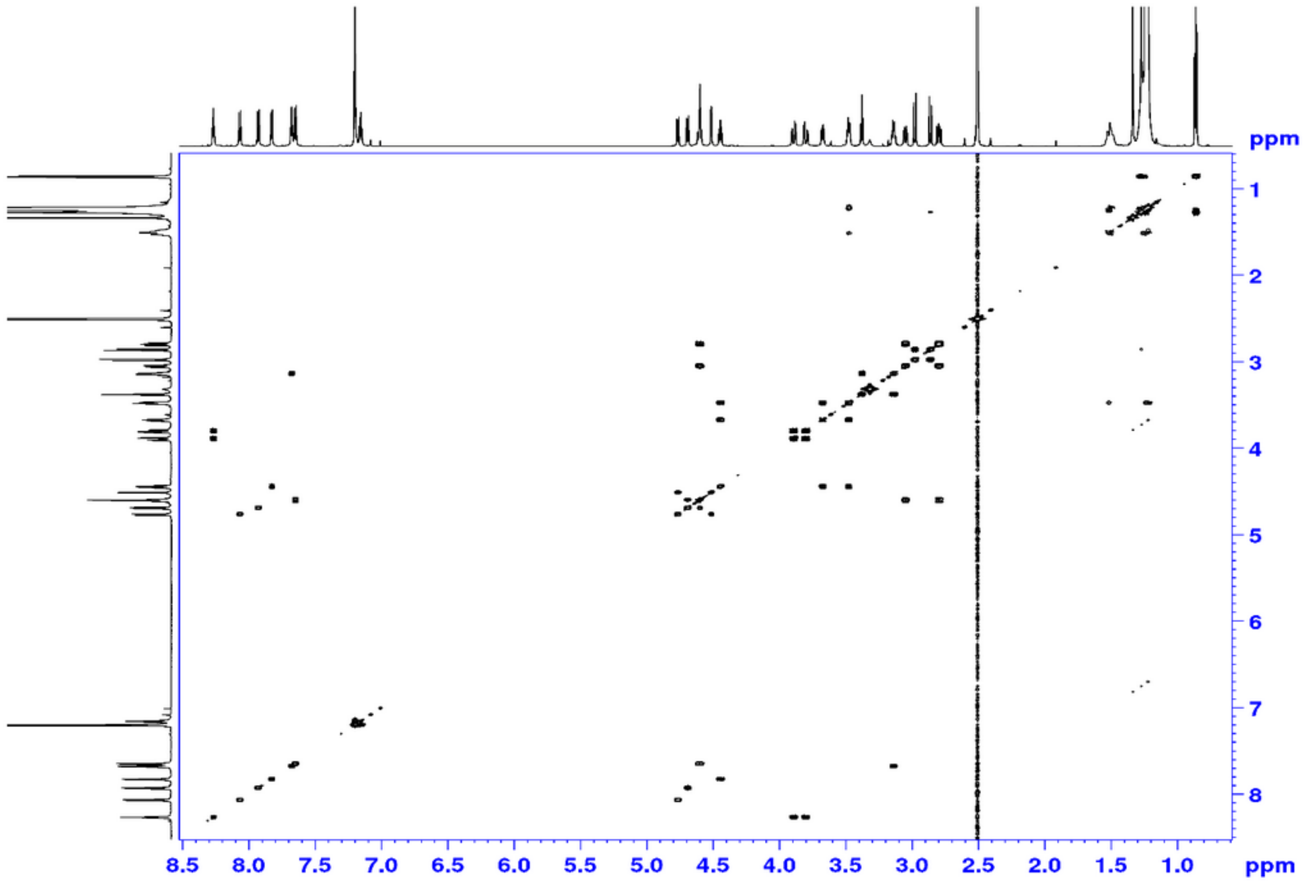


**Fig. S5:** COSY spectrum of cyanochelin B (700 MHz, DMSO-d6, 308K)

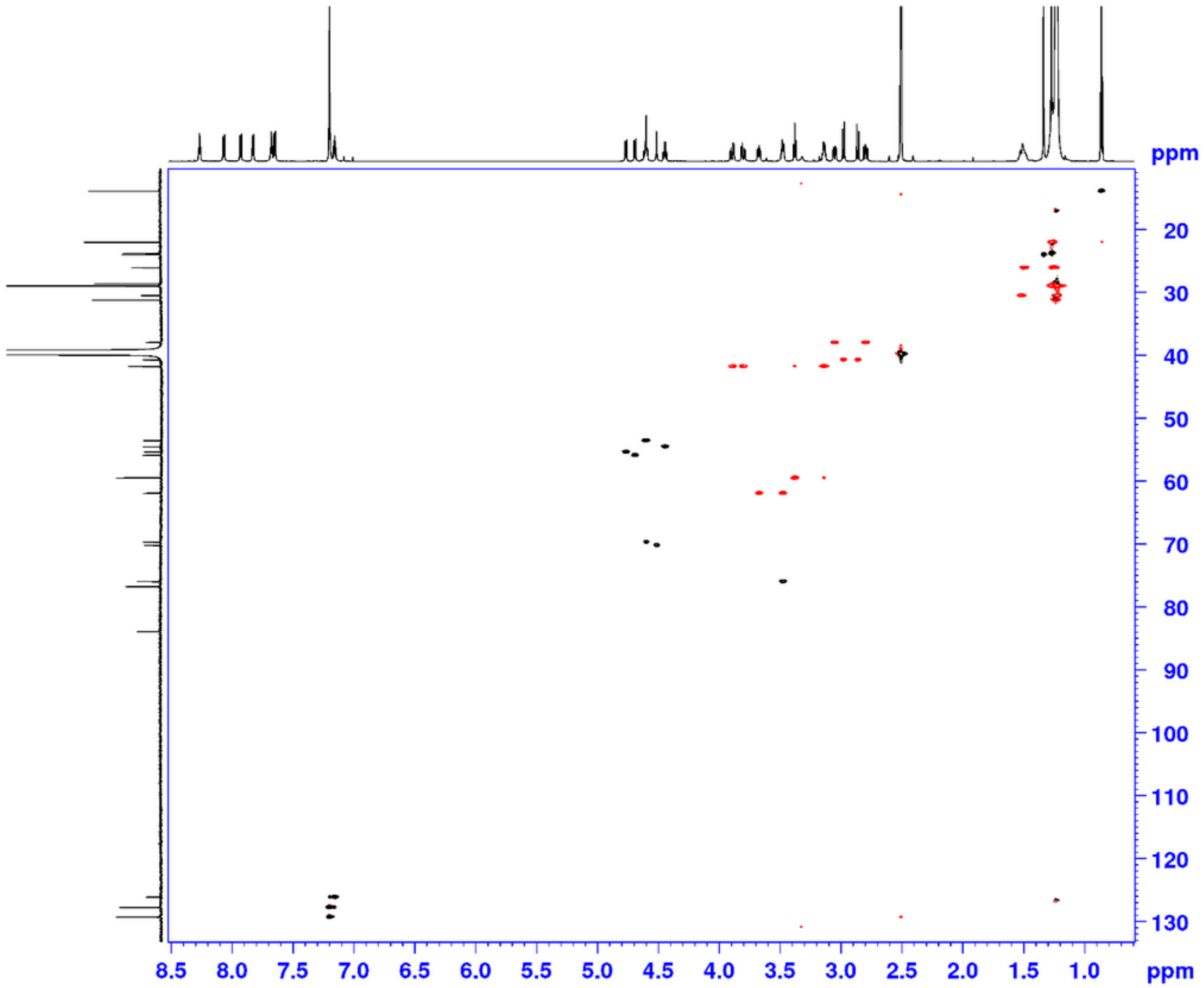


**Fig. S6:** HSQC spectrum of cyanochelin B (700 MHz, DMSO-d6, 308K)

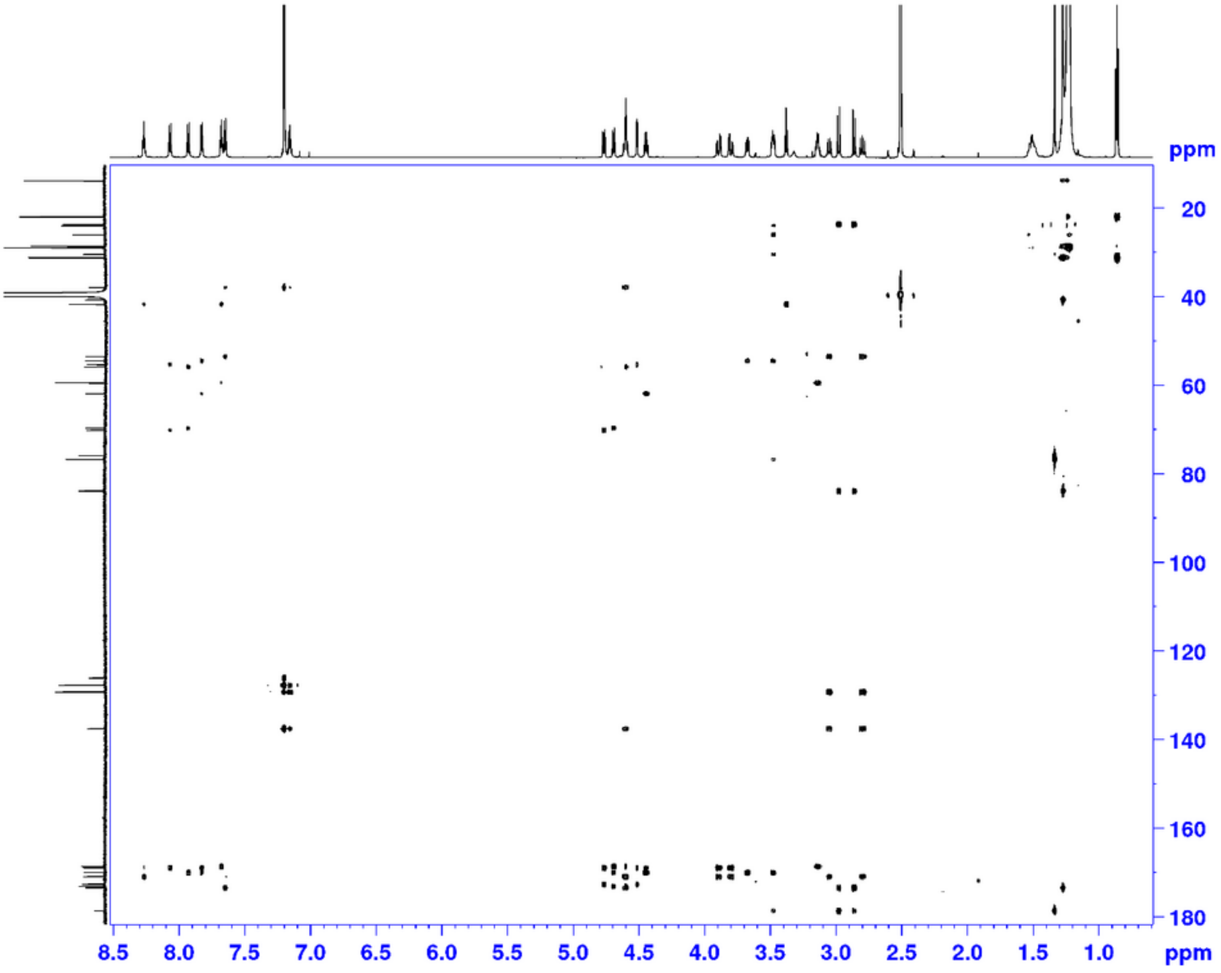


**Fig. S7:** HMBC spectrum of cyanochelin B (700 MHz, DMSO-d6, 308K)

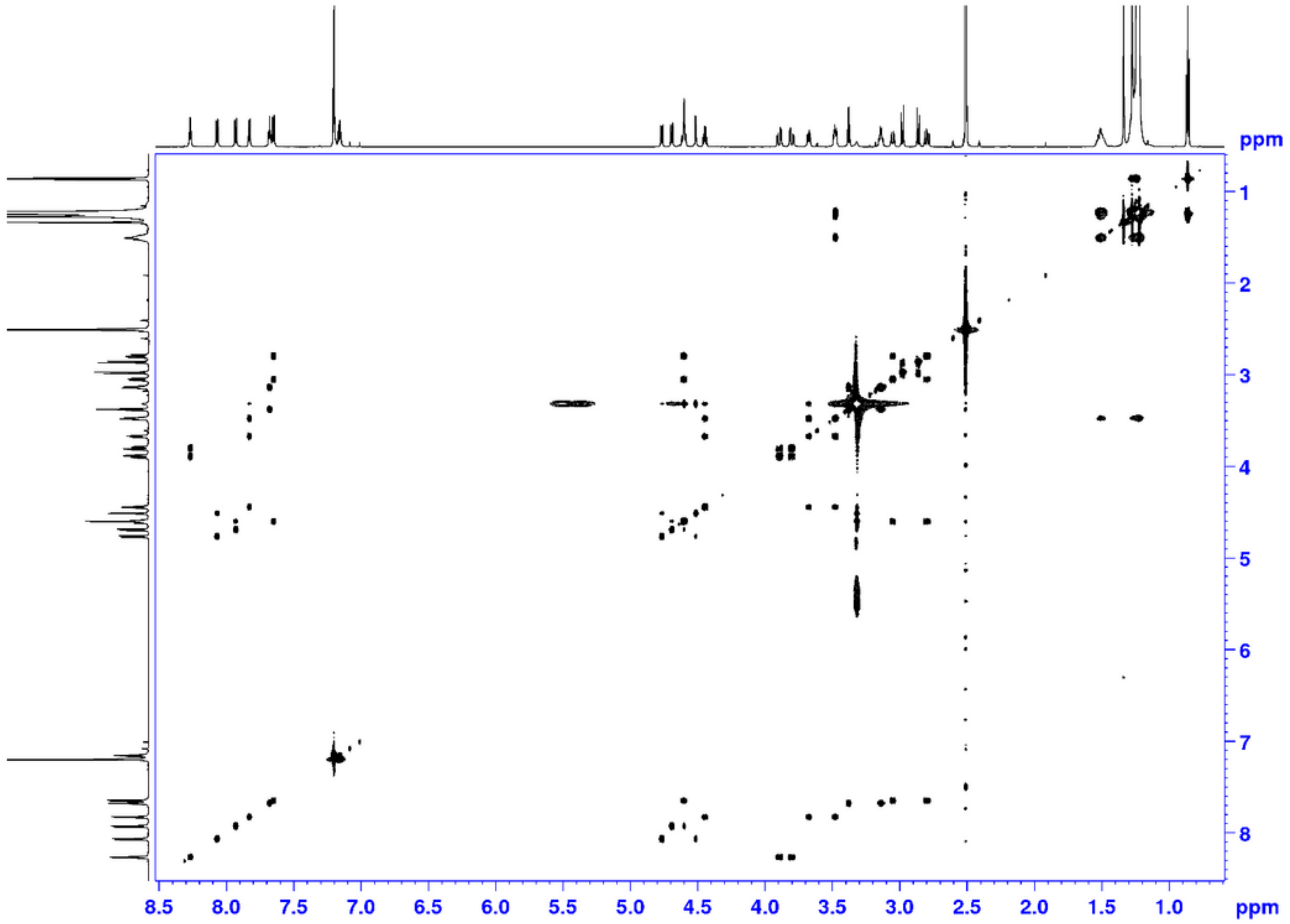


**Fig. S8:** TOCSY spectrum of cyanochelin B (700 MHz, DMSO-d6, 308K)

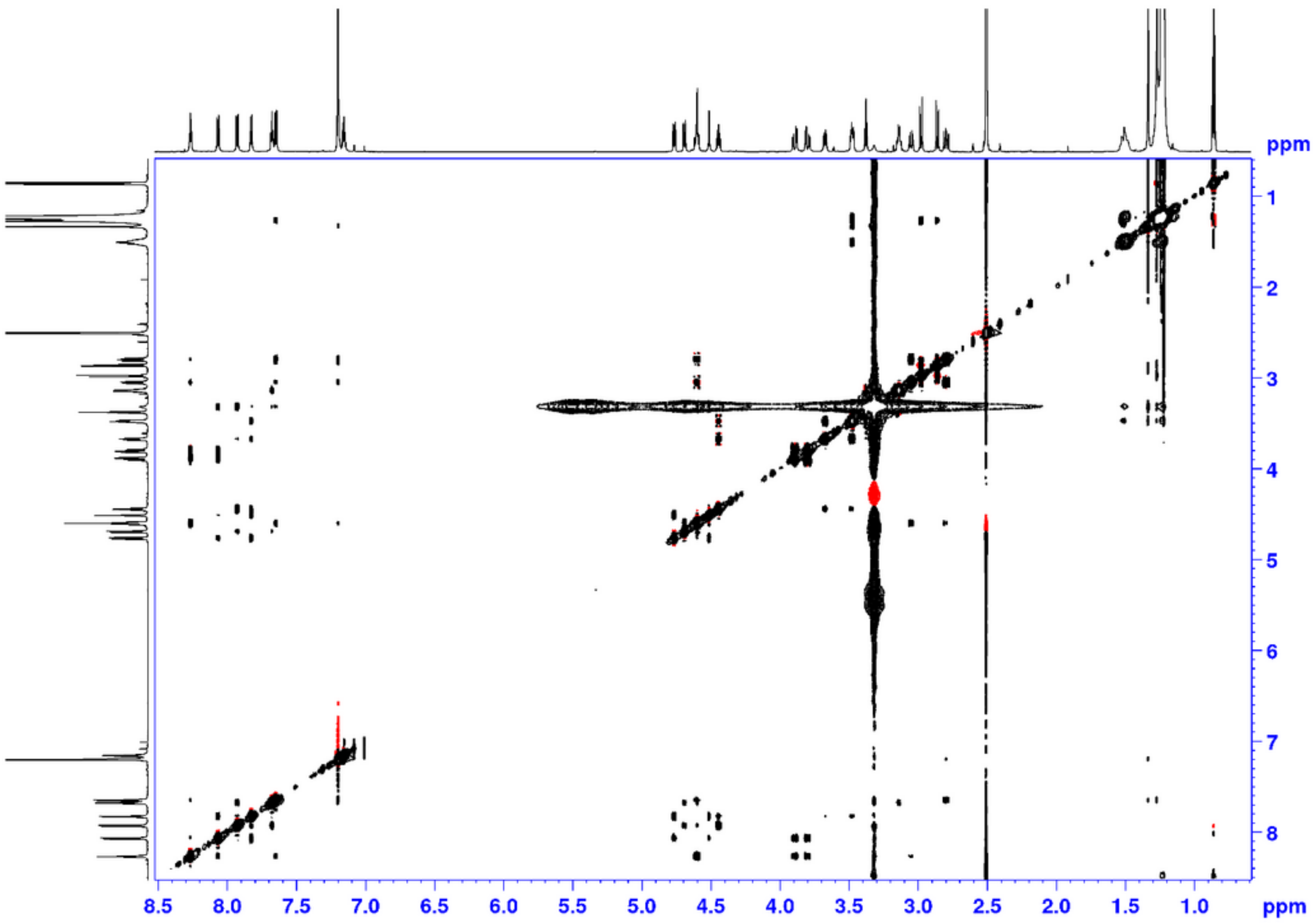


**Fig. S9:** NOESY spectrum of cyanochelin B (700 MHz, DMSO-d6, 308K)

A

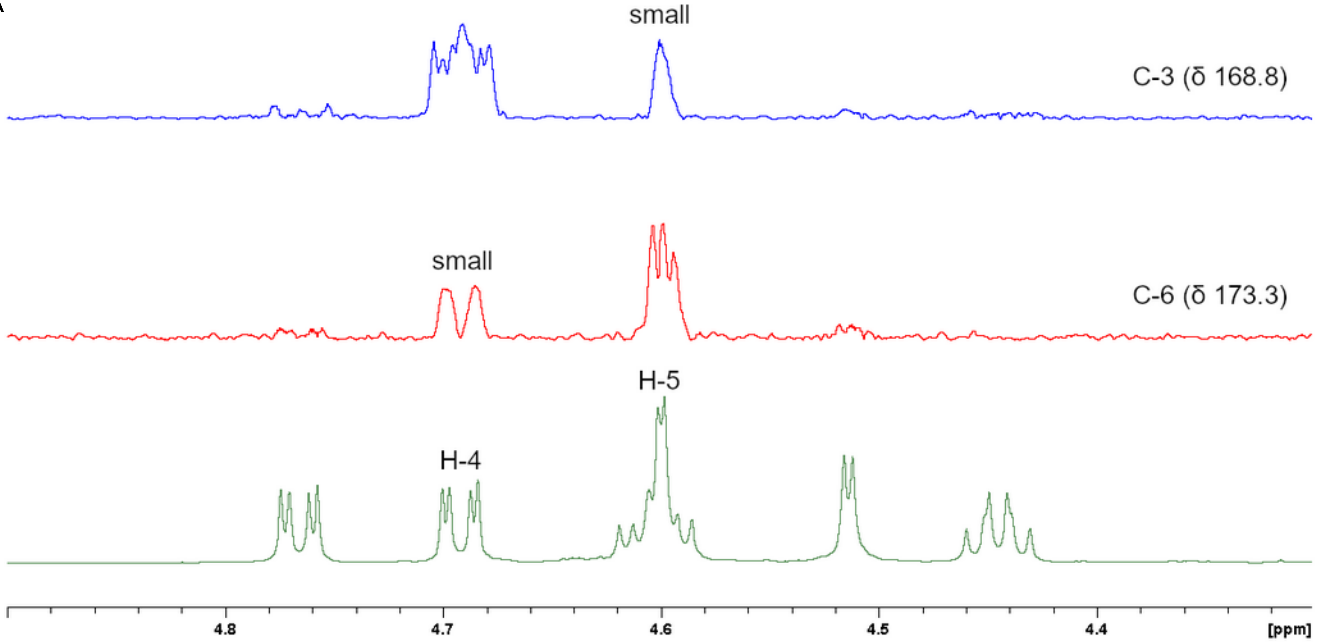


B

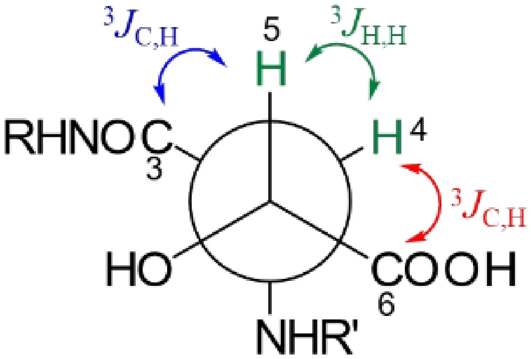


**Fig. S10:** (A) One-dimensional sections of the HMBC spectrum of cyanochelin B at δ 168.8 (C-3, blue trace) and δ 173.3 (C-6, red trace), compared with the standard $^{1}$H NMR spectrum (green trace). The HMBC spectrum was not $^{13}$C decoupled, so any $J_{C,H}$ would cause additional splitting of the relevant signal. The absence of any remarkable additional splitting showed that the $^{3}J_{C,H}$ between H-5 and C-3 and that between H-4 and C-6 (red trace) are both small. (B) Together with the small $^{3}J_{HH}$ between H-4 and H-5 (Table S3), these data showed gauche relationship between C-3 and H-5, H-4 and H-5, and H-4 and C-6, unequivocally determining the relative configuration of β-OH-Asp$^{6}$ as shown in the picture, i.e. *threo*.

A

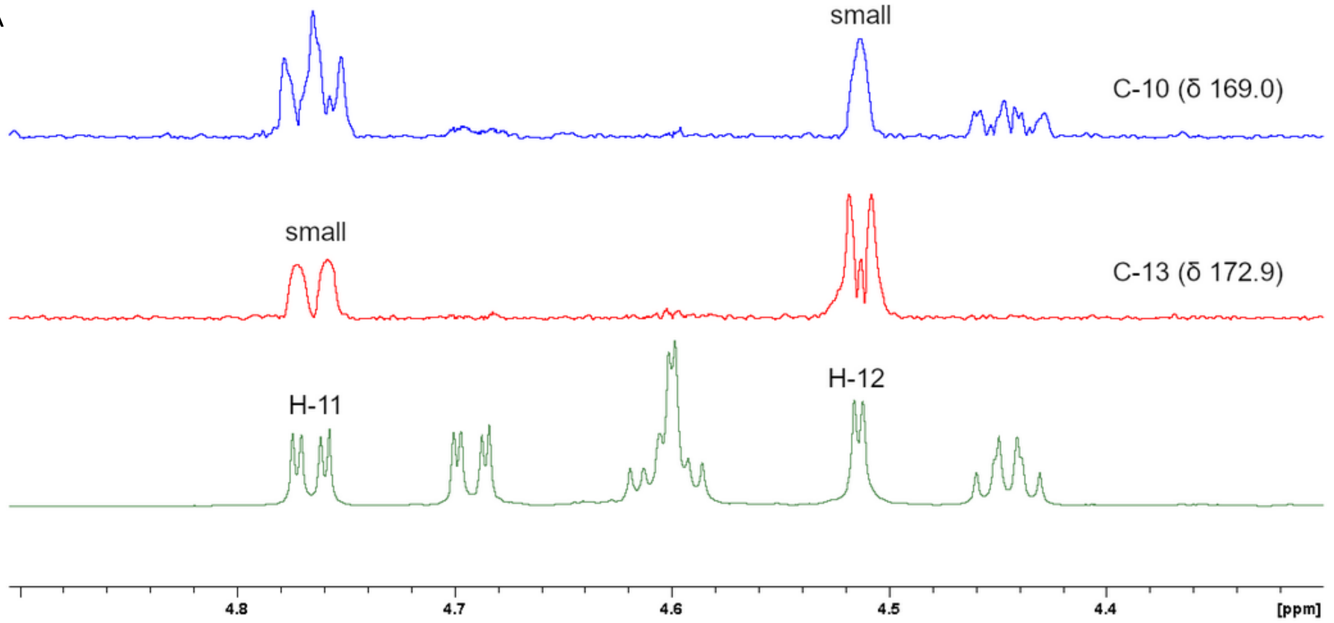


B

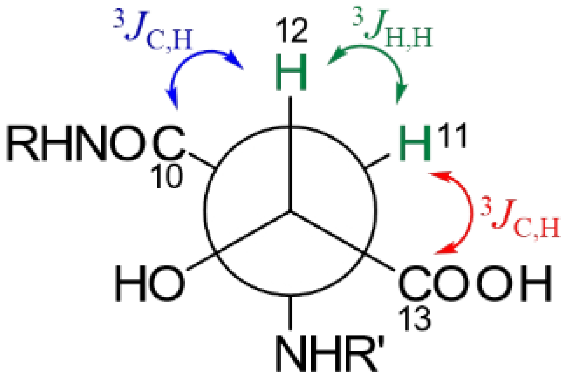


**Fig. S11:** (A) One-dimensional sections of the HMBC spectrum of cyanochelin B at δ 169.0 (C-10, blue trace) and δ 172.9 (C-13, red trace), compared with the standard $^{1}H$ NMR spectrum (green trace). The HMBC spectrum was not $^{13}C$ decoupled, so any $J_{C,H}$ would cause additional splitting of the relevant signal. The absence of any remarkable additional splitting shows that the $^{3}J_{C,H}$ between H-12 and C-10 and that between H-11 and C-13 (red trace) are both small. (B) Together with the small $^{3}J_{HH}$ between H-11 and H-12 (Table S3), these data showed gauche relationship between C-10 and H-12, H-10 and H-11, and H-10 and C-13, unequivocally determining the relative configuration of β-OH-Asp[4] as shown in the picture, i.e. *threo.*

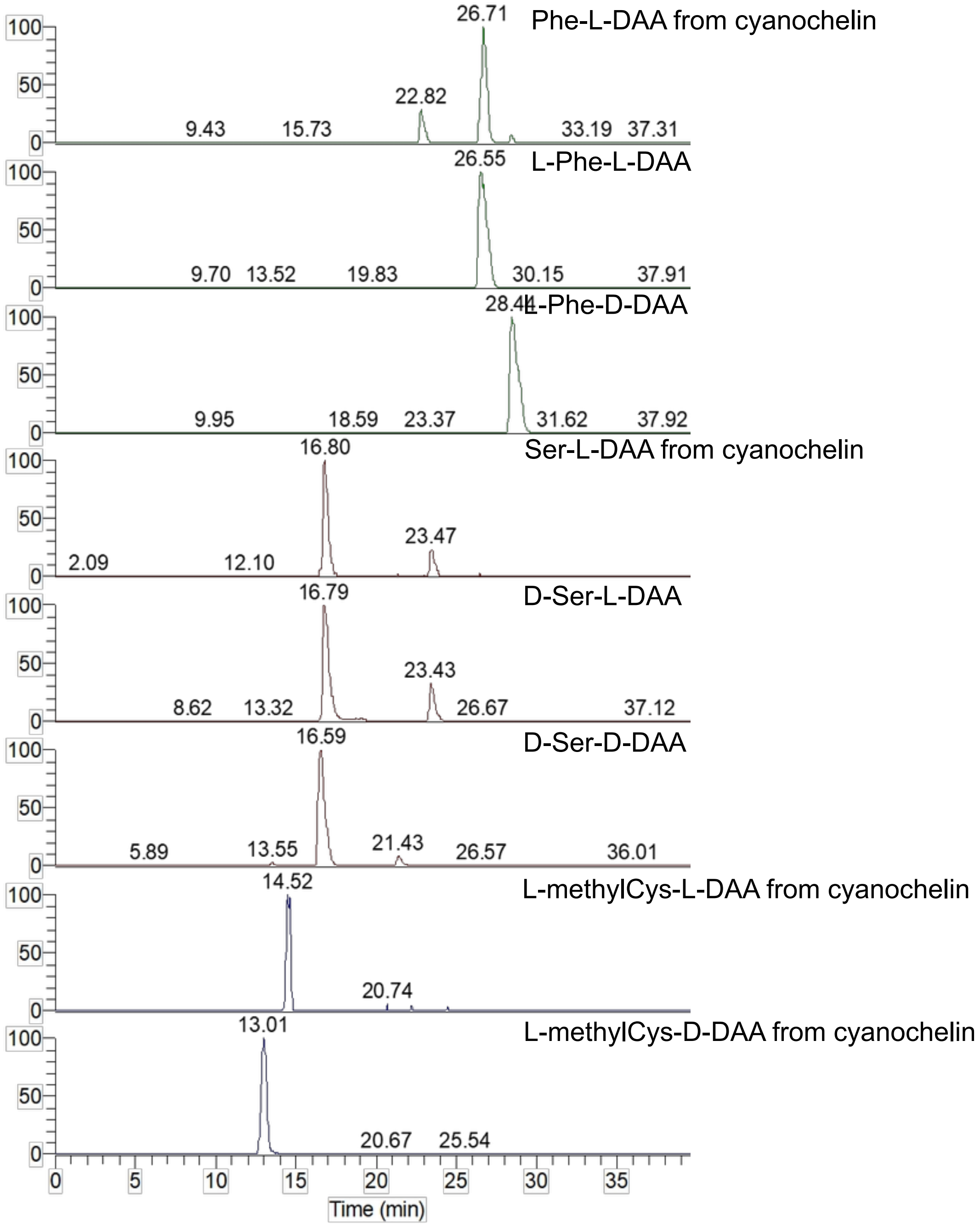


**Fig. S12:** LC-MS-enhanced Marfey's analysis of cyanochelin B. Extracted-ion chromatograms at *m/z* 418.1357 of the L-FDAA derivative from the hydrolysis of cyanochelin B and of D-and L-FDAA derivatives of L-Tyr (green trace). Extracted-ion chromatograms at *m/z* 358.0993 of the L-FDAA derivative from the hydrolysis of cyanochelin B and of D-and L-FDAA derivatives of D-Ser (red trace). Extracted-ion chromatograms at *m/z* 388.0921 of the D- and L-FDAA derivatives from the hydrolysis (blu trace).

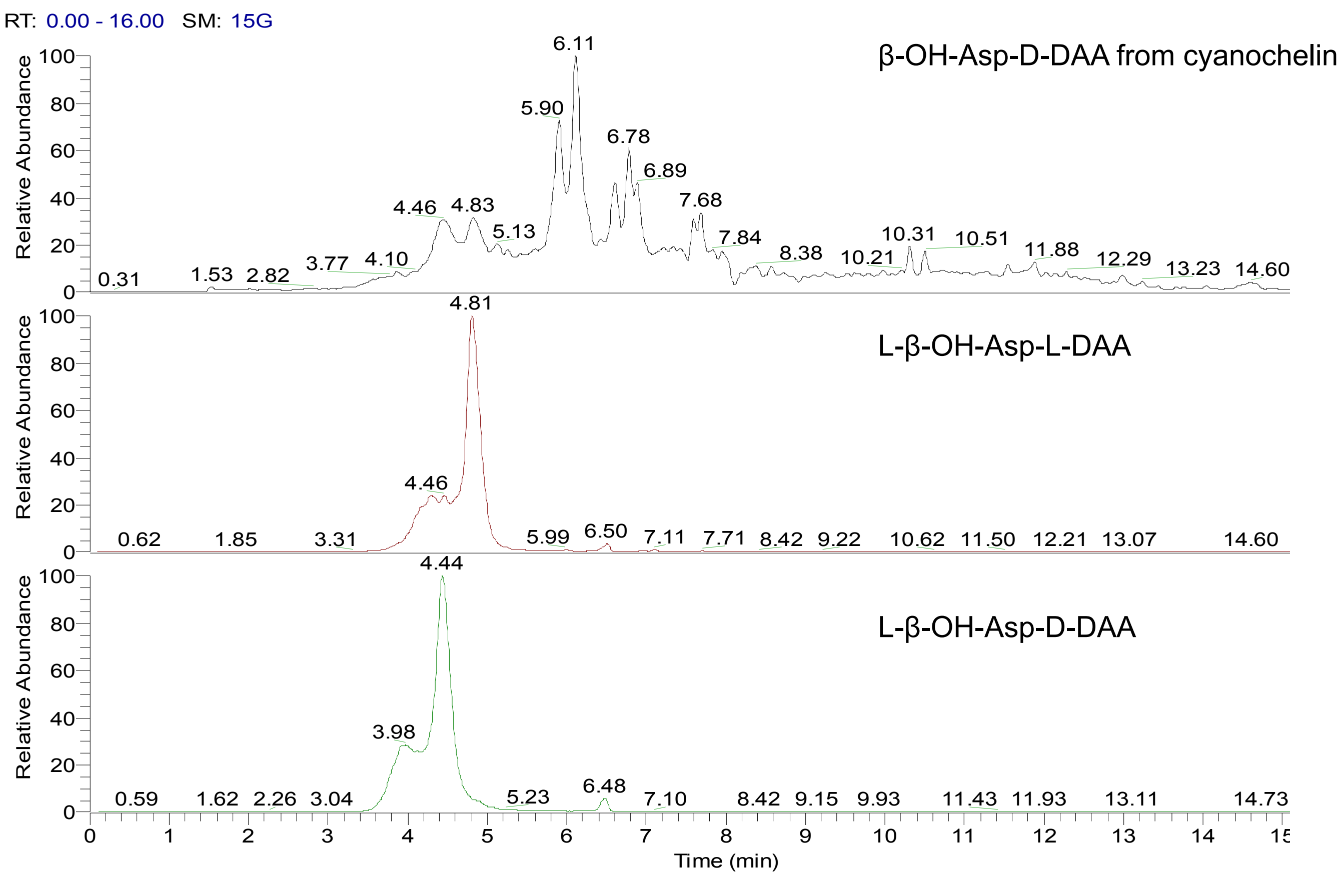


**Fig. S13:** LC-MS SRM-enhanced Marfey's analysis of cyanochelin B. In SRM, mass analyzers are set to a selected mass-to-charge ratio, focusing on specific precursor and product ions, the MS method involved two HRMS/MS scan events, following two product ions: m/z 356.1-358.1 and m/z 280.1-282.1 from the m/z precursor 402.2. Extracted-ion chromatograms at *m/z* 356.1-358.1 of the D-FDAA derivative from the hydrolysis of cyanochelin B (black trace) and of D- (green trace) and L-FDAA (red trace) derivatives L-β-OH-Asp.

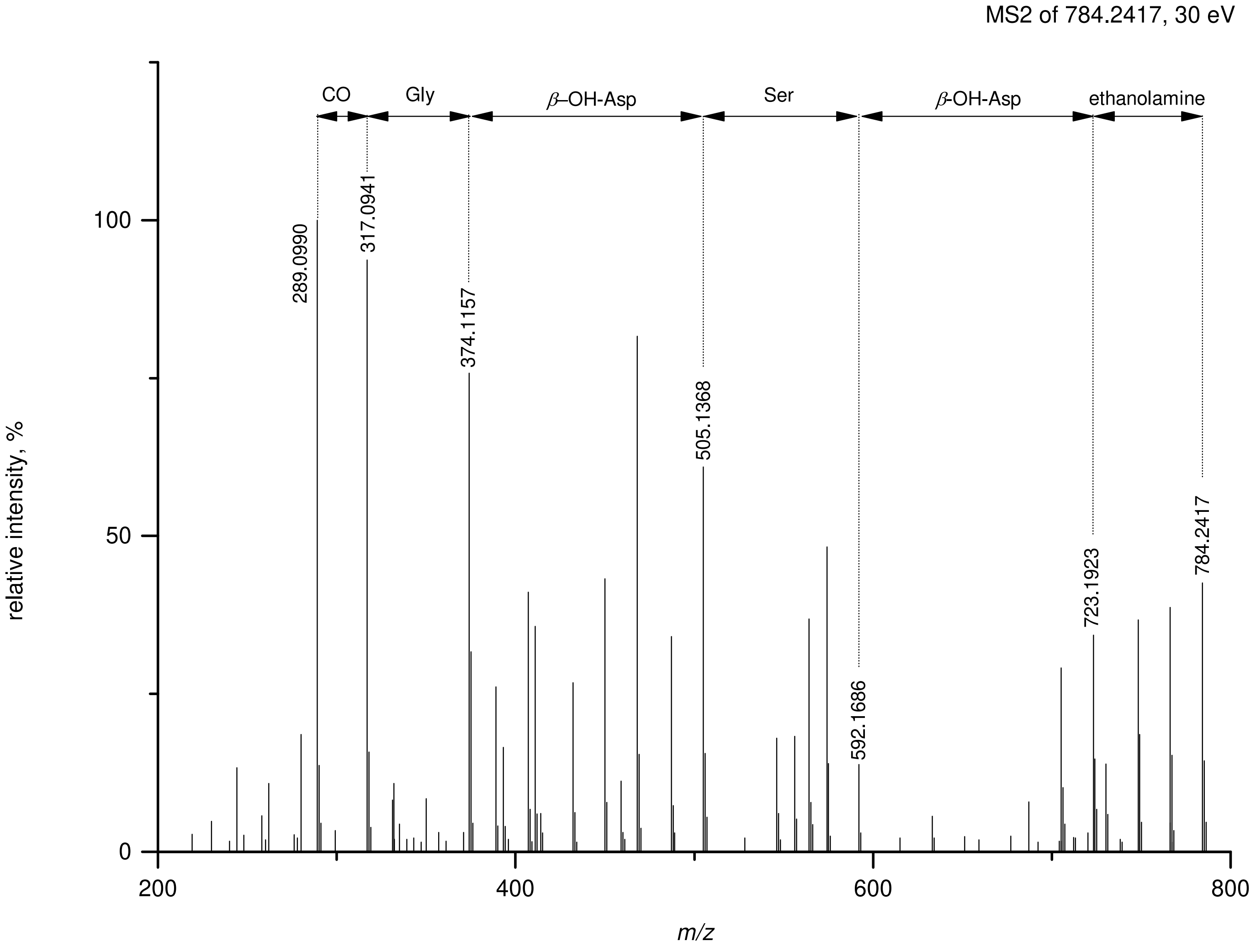


**Fig S14: Annotated MS/MS fragmentation spectrum of photolytic fragment *m/z* 784.2417 (PF1)**

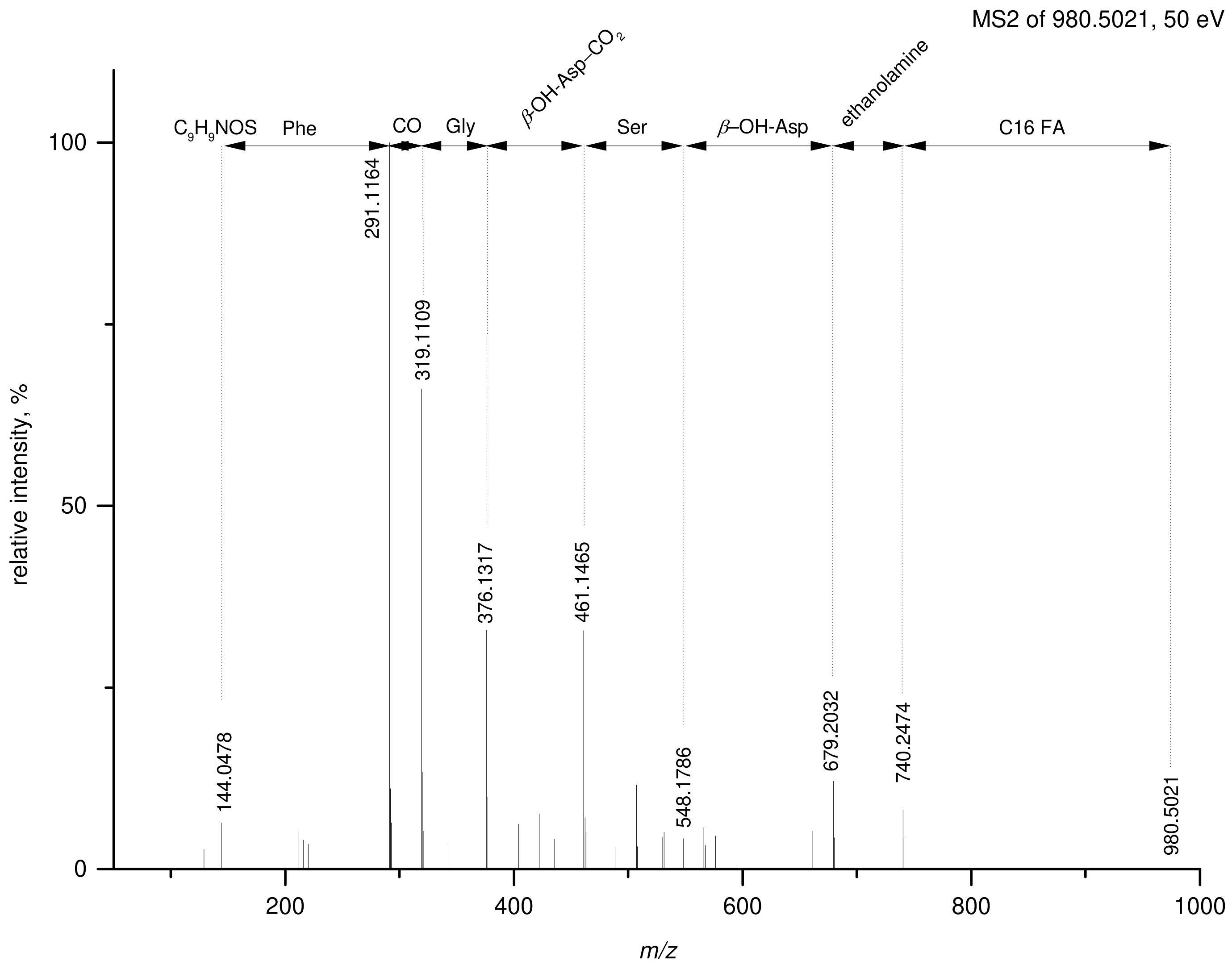


**Fig S15: Annotated MS/MS fragmentation spectrum of photolytic fragment *m/z* 980.5021 (PF2)**

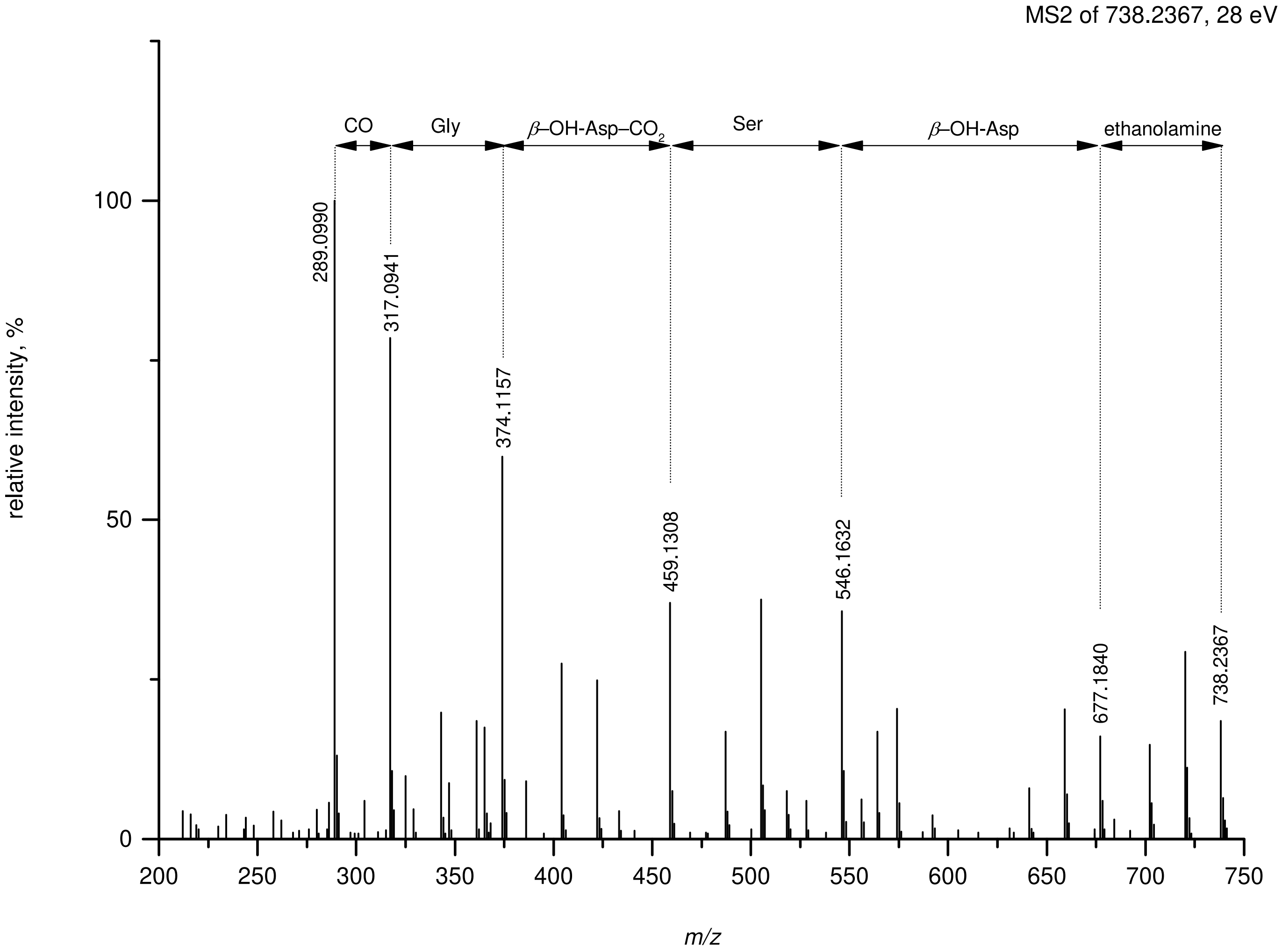


**Fig S16: Annotated MS/MS fragmentation spectrum of photolytic fragment 738.2367 (PF3)**

MS2 of 633.4073, 41 eV

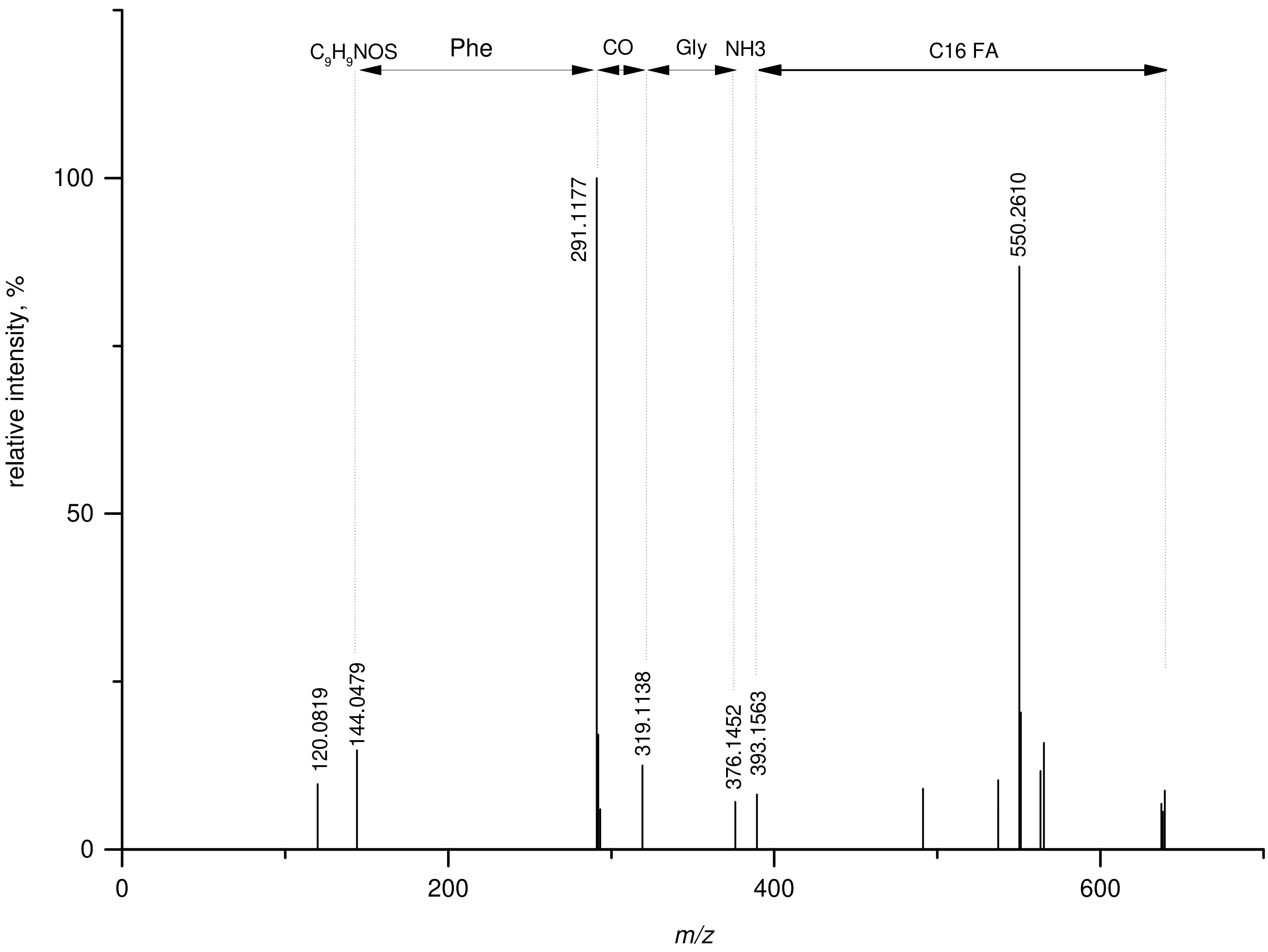


**Fig S17: Annotated MS/MS fragmentation spectrum of photolytic fragment 633.4073 (PF4)**

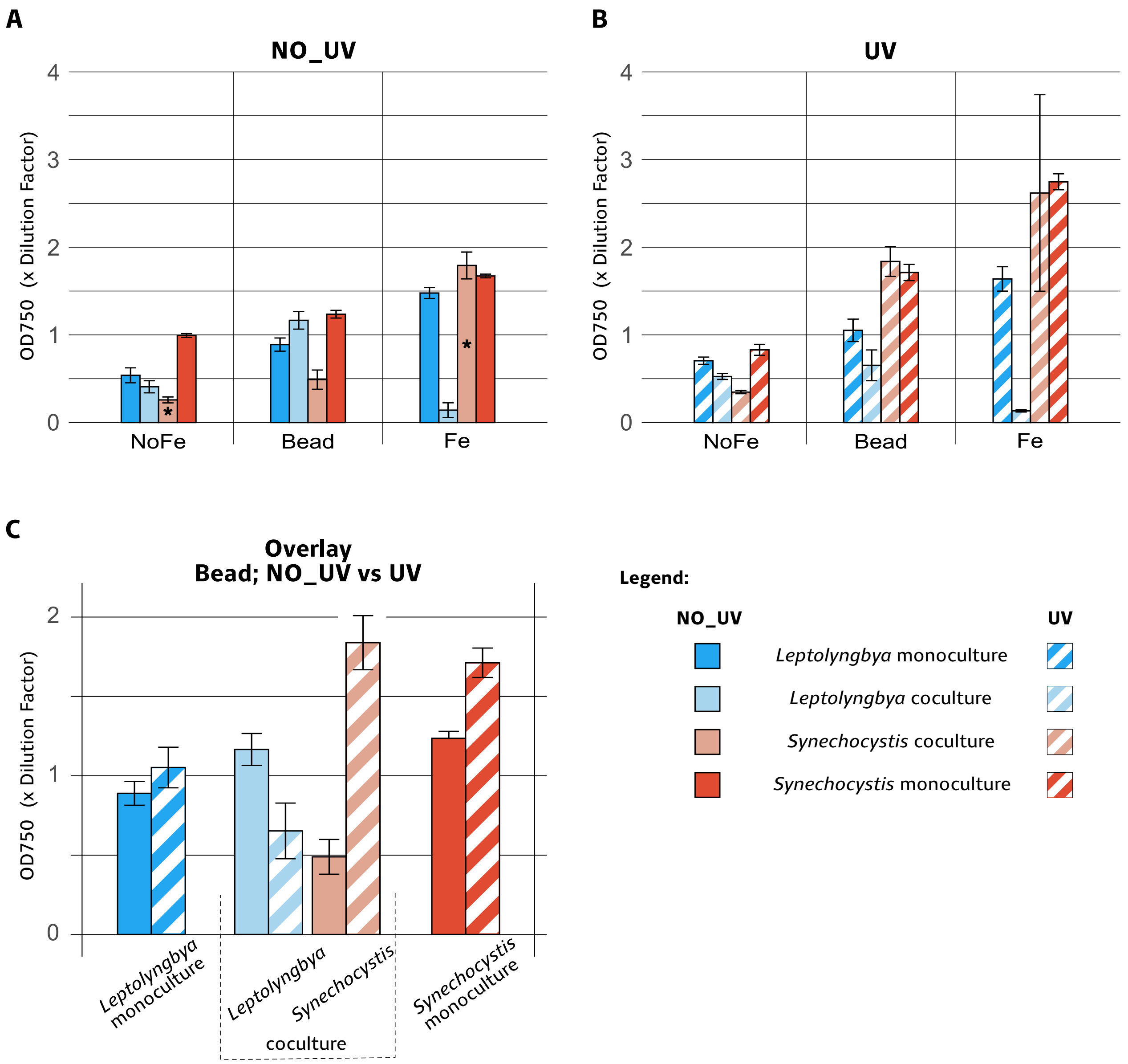


**Fig. S18:** A representative cultivation experiment showing the combined effects of UV-light and iron availability on culture density of Leptolyngbya and Synechocystis cultivated individually or combined in membrane separated compartments. Endpoint OD of Leptolyngbya and Synechocystis grown under different availability of iron: iron deprived media - NoFe; alginate-enclosed iron - Bead; standard BG-11 medium (Fe) in the absence of UV - **A)** or presence of UV - **B)**. Subfigure **C)** shows comparison of endpoint ODs of cultures grown with alginate-enclosed iron in the absence or presence of the UV-light. Asterisks indicate cultures where only two replicates were availabel for the given experiment run.

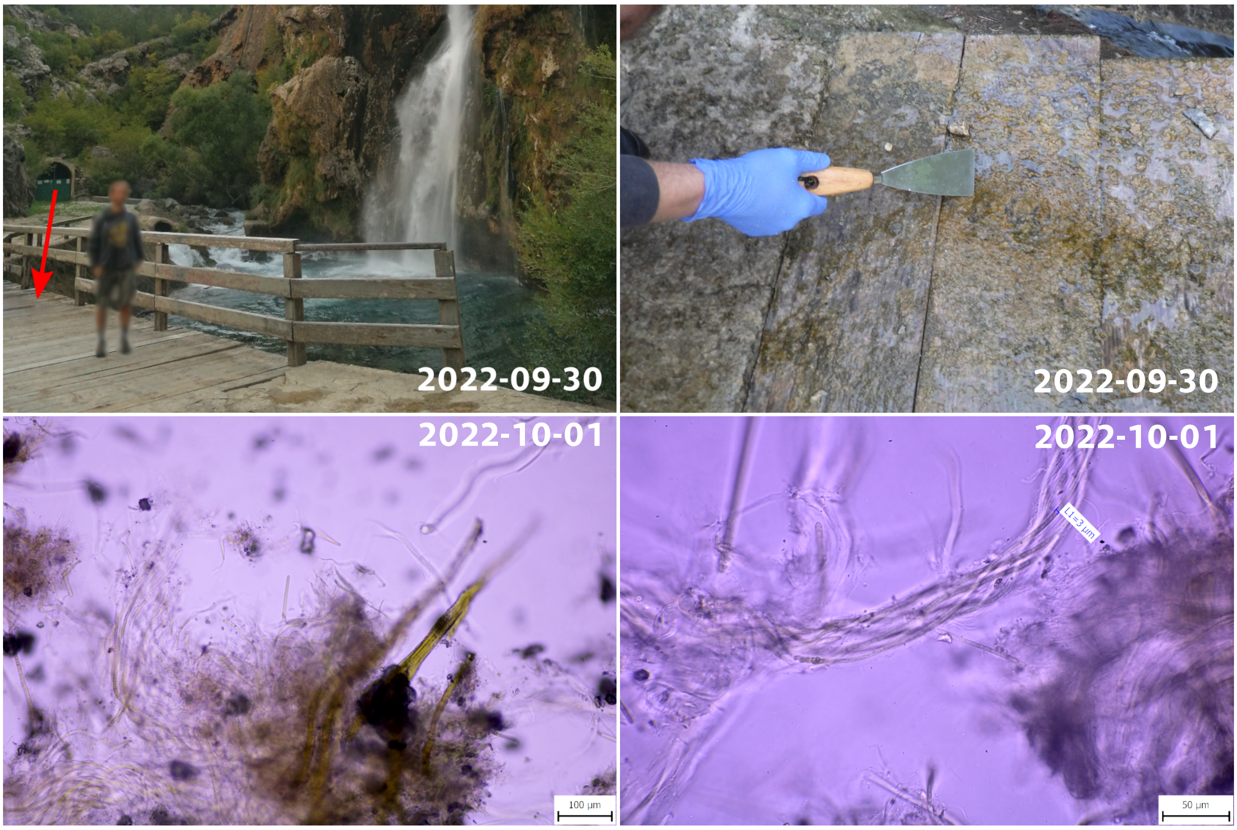


**Fig. S19: Field sample no. 146,** Incrustated mat on a bridge, GPS: N 44.04152, E O16.23488, altitude 87m

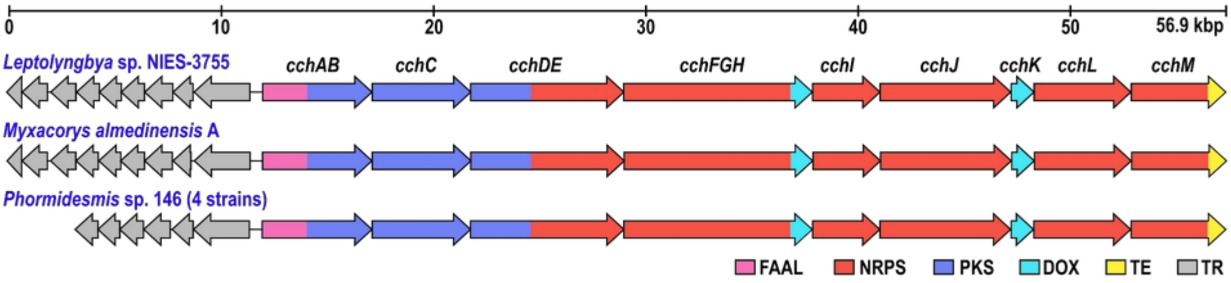


**Fig. S20:** Arrangement of the cyanochelin B biosynthetic gene clusters (BGCs) in strains of three separate genera of Leptolyngbyaceae (AP017310.1, WVIE00000000.1, JBJPHS000000000, JBJPHT000000000, JBJPHU000000000 and JBJPHV000000000). The NRPS/PKS biosynthetic core of all sequenced BGCs exhibits an identical topology and high average sequence identity (96%). Genes encoding siderophore transporters are found in opposite orientation adjacent to the first biosynthetic gene. FAAL - fatty acyl-AMP ligase; NRPS - non-ribosomal peptide synthetase; PKS - polyketide synthase; DOX - aspartate oxygenase; TE - thioesterase; TR - siderophore transport genes.

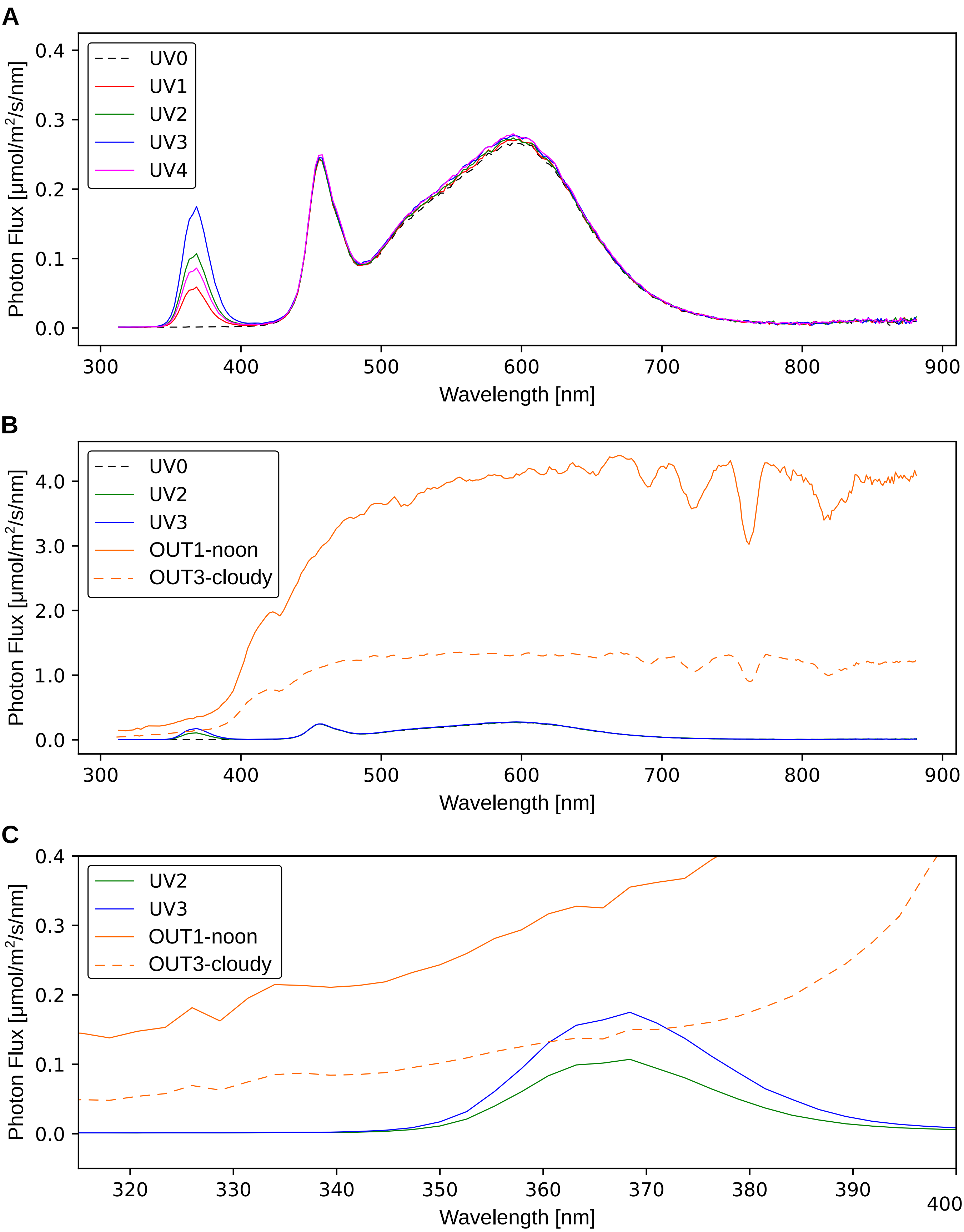


**Fig. S21:** Spectral characterisation of light conditions employed throughout the study. **A** Light conditions achieved by LED light sources in our cultivations and photolytic experiments. Light source of visible light was always on. Intensity of UV-A LED light source was adjusted by PWM regulator. UV0 - UV-A light source turned off, UV1 - UV-A light source at minimal settings, UV2 - UV-A light source at approximatelly half of the maximum, UV3 - UV-A light source at maximum output, UV4 - UV-A light source at half intentsity, measured under the lid that was used to cover the plates. **B,C** Comparison of the light conditions achieved in the lab to a representative measurements obtained outside the lab obtained around noon on 24th of March 2025, **C** - same as B, zoomed to 315-400 nm
